# Supplementary material for: Nrm1 is a bistable switch connecting cell cycle progression to transcriptional control
Source: EMBO Rep. 2025 Aug 29;26(20):5048–69. doi: 10.1038/s44319-025-00566-7 (PMC12550009; doi:10.1038/s44319-025-00566-7)
Supplement: Supplementary file 1 — Table EV1 [file 44319_2025_566_MOESM1_ESM.docx]

| **Table EV1. Strains used in this work** | |
| --- | --- |
| **Strain** | **Genotype** |
| WT | *972 h-* |
| CS16 | *h- cdc25-22* |
| JA195 | *h- wee1-50 leu1-32* |
| JA777 | *h+ yox1-13Myc-KanMX6* |
| JA780 | *h? cdc25-22 yox1-13xMyc-KanMX6* |
| JA795 | *h- yox1Δ::KanMX6* |
| JA804 | *h- rad3Δ::KanMX6* |
| JA940 | *h+ cdc25-22 mik1Δ::KanMX6 yox1Δ::ura leu1-32 ura4-D18* |
| JA977 | *h+ yox1-13xMyc-KanMX6 nrm1-3xHA-NatMX6* |
| JA1106 | *h- nrm1Δ::BleMX6* |
| JA1173 | *h- cdc25-22 nrm1-3xHA-NatMX6 leu1-32* |
| JA1174 | *h- cdc25-22 rep2-TAP-KanMX6 leu1-32* |
| JA1176 | *h- nrm1-3xHA-NatMX6* |
| JA1201 | *h- res2-roGFP2:NatMX6* |
| JA1202 | *h+ yox1Δ::ura4+ res2-yox1-Nat+ ura4-D18* |
| JA1203 | *h- nrm1Δ::kan+ res2-nrm1-Nat+* |
| JA1207 | *h+ cdc25-22 yox1Δ::ura4+ res2-yox1-NatMX6 ura4-D18* |
| JA1242 | *h+ yox1Δ::KanMX6 nrm1Δ::BleMX6* |
| JA1344 | *h+ mts3-1 nrm1-3xHA-NatMX6 leu1-32* |
| JA1512 | *h- rep2Δ::KanMX6* |
| JA1731 | *h+ nda3-KM311 nrm1-3xHA-NatR+* |
| JA1767 | *h+ wee1-50 res2-nrm1:NatMX6 nrm1Δ::BleMX6* |
| JA1780 | *h+ wee1-50 res2-yox1:NatMX6 yox1Δ::KanRMX6* |
| JA1784 | *h+ wee1-50 res2-roGFP2-NatMX6* |
| JA1785 | *h- nrm1-3A-3xHA-KanMX6 (T9A S57A T287A) ura4-D18* |
| JA1786 | *h- nrm1-4A-3xHA-KanMX6 (T9A S57A T116A T287A) ura4-D18* |
| JA1787 | *h- nrm1-5A-3xHA-KanMX6 (T9A S57A S237A T241 T287A) ura4-D18* |
| JA1792 | *h+ nrm1-6A-3xHA-KanMX6 (T9A S57A T116A S237A T241A T287A) ura4-D18* |
| JA1879 | *h+ yox1Δ::NatMX6* |
| JA2003 | *h- rep2Δ::KanMX6 yox1Δ::NatMX6* |
| JA2033 | *h- rep2Δ::KanMX6 res2-linker-rep2-3HA-NatMX6* |
| JA2054 | *h+ cdc2-asM17-BsdMX6* |
| JA2080 | *h+ cdc2-asM17-BsdMX6 rep2Δ::KanMX6* |
| JA2082 | *h+ cdc2-asM17-BsdMX6 rep2Δ::KanMX6 yox1Δ::NatMX6* |
| JA2112 | *h- nrm1-SA-3xHA-KanMX6 (T9A T40A S57A T116A S155A S206A S237A T241A T272A T287A T307A) ura4-D18* |
| JA2113 | *h- nrm1-SD-3xHA-KanMX6 (T9E T40E S57D T116E S155D S206D S237D T241E T272E T287D T307E) ura4-D18* |
| JA2114 | *h+ nda3-KM311 nrm1-SA-3xHA-KanMX6* |
| JA2115 | *h+ nda3-KM311 nrm1-SD-3xHA-KanMX6* |
| JA2176 | *h- cdc25-22 nrm1-SA-3xHA-KanMX6* |
| JA2179 | *h- cdc25-22 nrm1-SD-3xHA-KanMX6* |
| JA2197 | *h+ pSty1-HA-mRFP (int @ leu1) leu1-32 rep2Δ::KanMX6 yox1Δ::NatMX6* |
| JA2218 | *h- pSty1-HA-mYFP (int @ leu1) leu1-32* |
| JA2223 | *h+ cdc2-asM17-BsdMX6 mik1Δ::KanMX6 yox1Δ::NatMX6* |
| JA2231 | *h- wee1-50 rep2::KanMX6 res2-linker-rep2-3HA-NatMX6* |
| JA2267 | *h? nrm1-SA-3xHA-KanMX6 yox1-13xMyc-NatMX6* |
| JA2268 | *h? nrm1-SD-3xHA-KanMX6 yox1-13xMyc-NatMX6* |
| JA2299 | *h? nrm1-SA-3HA-KanMX6 leu1-32* |
| JA2417 | *h? nrm1-SD-3HA-KanMX6 leu1-32* |
| JA3345 | *h- rad52-mNG-NatMX6* |
| JA3500 | *h- cdc25-22 nrm1-3xHA-MX6* |
| JA3508 | *h- his5::act1p-nrm1-A2-NLS-sfGFP-NatMX6:his5+* |
| JA3510 | *h- his5::act1p-nrm1-C2-NLS-sfGFP-NatMX6:his5+* |
| JA3571 | *h- his5::act1p-nrm1-A2-NLS-sfGFP-NatMX6:his5+ ubc4.P61-* |
| JA3572 | *h- his5::act1p-nrm1-C2-NLS-sfGFP-NatMX6:his5+ ubc4.P61S* |
| JA3573 | *h- his5::act1p-nrm1-A2-NLS-sfGFP-NatMX6:his5+ ubc11.P93L* |
| JA3574 | *h- his5::act1p-nrm1-C2-NLS-sfGFP-NatMX6:his5+ ubc11.P93L* |
| JA3603 | *h- his5::act1p-nrm1-A2-NLS-sfGFP-NatMX6:his5+ ubc4.P61S ubc11.P93L* |
| JA3604 | *h- his5::act1p-nrm1-C2-NLS-sfGFP-NatMX6:his5+ ubc4.P61S ubc11.P93L* |
| JA3658 | *h- his5::act1p-nrm1-FL-NLS-sfGFP-NatMX6:his5+* |
| JA3659 | *h- his5::act1p-nrm1-FL-NLS-sfGFP-NatMX6:his5+ ubc4.P61S* |
| JA3660 | *h- his5::act1p-nrm1-FL-NLS-sfGFP-NatMX6:his5+ ubc11.P93L* |
| JA3661 | *h- his5::act1p-nrm1-FL-NLS-sfGFP-NatMX6:his5+ ubc4.P61S ubc11.P93L* |
| JA3673 | *h? nrm1-3xHA-NatMX6 ura4::pcn1p-eGFP-pcn1-nmt1ter-NatMX6:ura4+ ura4-D18* |
| JA3674 | *h? nrm1-3xHA-NatMX6 ura4::pcn1p-mCherry-pcn1-nmt1ter-NatMX6:ura4+ ura4-D18* |
| JA3675 | *h? nrm1-SA-3xHA-KanMX6 ura4::pcn1p-eGFP-pcn1-nmt1ter-NatMX6:ura4+ ura4-D18* |
| JA3676 | *h? nrm1-SA-3xHA-KanMX6 ura4::pcn1p-mCherry-pcn1-nmt1ter-NatMX6:ura4+ ura4-D18* |
| JA3677 | *h? nrm1-SD-3xHA-KanMX6 ura4::pcn1p-eGFP-pcn1-nmt1ter-NatMX6:ura4+ ura4-D18* |
| JA3678 | *h? nrm1-SD-3xHA-KanMX6 ura4::pcn1p-mCherry-pcn1-nmt1ter-NatMX6:ura4+ ura4-D18* |
| JA3702 | *h? nrm1-3xHA-NatMX6 sid2-GFP:ura4+ lys3::pcn1p:mCherry-pcn1:lys3+ ade6::act1p:mCherry-RitC:ade6+ leu1::eno101p-SynCut3-mTagBFP2:leu1+ lys3-D20 ade6-D19 leu1-32 ura4-D18?* |
| JA3703 | *h? nrm1-SA-3xHA-KanMX6 sid2-GFP:ura4+ lys3::pcn1p:mCherry-pcn1:lys3+ ade6::act1p:mCherry-RitC:ade6+ leu1::eno101p-SynCut3-mTagBFP2:leu1+ lys3-D20 ade6-D19 leu1-32 ura4-D18?* |
| JA3704 | *h? nrm1-SD-3xHA-KanMX6 sid2-GFP:ura4+ lys3::pcn1p:mCherry-pcn1:lys3+ ade6::act1p:mCherry-RitC:ade6+ leu1::eno101p-SynCut3-mTagBFP2:leu1+ lys3-D20 ade6-D19 leu1-32 ura4-D18?* |
| JA3790 | *h- rad3::kanMX6 rad52-mNG-NatMX6+* |
| JA3799 | *h- ade6::pCMV-tetR-enotetSW2p-nrm1-3HA-adh1ter-HphMX:ade6+* |
| JA3800 | *h- yox1Δ::KanMX6 ade6::CMVp-tetR-enotetSW2p-nrm1-3HA-adh1ter-HphMX:ade6+* |
| JA3801 | *h- nrm1Δ::BleMX6 ade6::pCMV-tetR-enotetSW2p-nrm1-3HA-adh1ter-HphMX:ade6+* |
| JA3802 | *h+ yox1Δ::KanMX6 nrm1Δ::phleo ade6::pCMV-tetR-enotetSW2p-nrm1-3HA-adh1ter-HphMX:ade6+* |
| JA3806 | *h- nrm1-3xHA-Nat rad52-mNG-KanR+* |
| JA3807 | *h- nrm1-SA-3xHA-KanMX6 rad52-mNG-NatMX6* |
| JA3808 | *h- nrm1-SD-3xHA-KanMX6 rad52-mNG-KanMX6* |
| JA3810 | *h- ade6::CMVp-tetR-enotetSW2p-yox1-Myc-adh1ter-hphMX6:ade6+* |
| JA3811 | *h- yox1Δ::KanMX6 ade6::CMVp-tetR-enotetSW2p-yox1-Myc-adh1ter-hphMX6:ade6+* |
| JA3812 | *h- nrm1Δ::BleMX6 ade6::CMVp-tetR-enotetSW2p-yox1-Myc-adh1ter-hphMX6:ade6+* |
| JA3813 | *h- yox1Δ::KanMX6 nrm1Δ::BleMX6 ade6::CMVp-tetR-enotetSW2p-yox1-Myc-adh1ter-hphMX6:ade6+* |
