# Supplementary material for: Nrm1 is a bistable switch connecting cell cycle progression to transcriptional control
Source: EMBO Rep. 2025 Aug 29;26(20):5048–69. doi: 10.1038/s44319-025-00566-7 (PMC12550009; doi:10.1038/s44319-025-00566-7)
Supplement: Supplementary file 2 — Table EV2 [file 44319_2025_566_MOESM2_ESM.docx]

| **Table EV2. Plasmids used in this work** | |
| --- | --- |
| **Number** | **Description** |
| pAY584 | pREP3x-srw1 |
| pAY610 | pRSET-A-Nrm1 |
| pAY832 | pREP3x-slp1 |
| pAY833 | pREP3x-skp1 |
| pAY984 | nmt41x-Nrm1-A2-NLS-GFP episomal |
| pAY986 | nmt41x-Nrm1-C2-NLS-GFP episomal |
| pAY1092 | nmt41x-Nrm1-A2 (KEN A)-NLS-GFP (episomal) |
| pAY1100 | nmt41x-Nrm1 FL-NLS-GFP episomal |
| pAY1101 | nmt41x-Nrm1-FL (KEN A)-NLS-GFP episomal |
| pAY1114 | pREP4 - 6His-Ubi |
| pAY1149 | nmt41x-Nrm1-FL-SA-NLS-GFP episomal |
| pAY1150 | nmt41x-Nrm1-FL-SD-NLS-GFP episomal |
| pAY1198 | nmt41x-Nrm1-FL (DBox A)-NLS-GFP episomal |
| pAY1274 | pHis5StuI act1p-Nrm1-A2-NLS-sfGFP-nmt1ter-NatMX6 |
| pAY1276 | pHis5StuI act1p-Nrm1-C2-NLS-sfGFP-nmt1ter-NatMX6 |
| pAY1290 | nmt41x-Nrm1-C2 (251-255 + 266-270 A)-NLS-GFP episomal |
| pAY1294 | pLSB-Kan ubc4.P61S sgRNA |
| pAY1295 | pLSB-Hph ubc11.P93L sgRNA |
| pAY1361 | pHis5StuI act1p-Nrm1-FL2-NLS-sfGFP-nmt1ter-NatMX6 |
| pAY1362 | nmt41x-Nrm1-C2 (H252A W255A R263A V265A L267A mut)-NLS-GFP episoma |
| pAY1363 | nmt41x-Nrm1-A2 (DBox A)-NLS-GFP episomal |
| pAY1364 | nmt41x-Nrm1-A2 (KEN DBox A)-NLS-GFP episomal |
| pAY1365 | nmt41x-Nrm1-FL (KEN DBox A)-NLS-GFP episomal |
| pAY1366 | nmt41x-Nrm1-FL (KEN DBox 251-255 + 266-270 A)-NLS-GFP episomal |
| pAY1367 | nmt41x-Nrm1-FL (KEN DBox H252A W255A R263A V265A L267A A)-NLS-GFP episomal |
| pAY1410 | pAde6NotI-pCMV-tetR-PenotetSW2-Nrm1-3HA-ADH1ter-HphMX |
| pAY1411 | pRSET-A-Nrm1-SA |
| pAY1412 | pAde6NotI-pCMV-tetR-PenotetSW2-Yox1-1xMyc-ADH1ter-HphMX |
