# Supplementary material for: Nrm1 is a bistable switch connecting cell cycle progression to transcriptional control
Source: EMBO Rep. 2025 Aug 29;26(20):5048–69. doi: 10.1038/s44319-025-00566-7 (PMC12550009; doi:10.1038/s44319-025-00566-7)
Supplement: Supplementary file 3 — Table EV3 [file 44319_2025_566_MOESM3_ESM.docx]

| **Table EV3. Primers used in this work** | |  |
| --- | --- | --- |
| **Oligo** | **Sequence** | **Used for** |
| JA488 | TTTGTCAAAAAGTTTTCCCACATAC | Intergenic Region Forward |
| JA489 | GCGGTTTCCCTTTCTAAGAGACT | Intergenic Region Reverse |
| JA643 | TTAATTAGATTTCGCGTTTACGTGT | *cdc18* prom ChIP forward |
| JA644 | ATTAACTTCGTGCTTGGTGTTTAAG | *cdc18* prom ChIP reverse |
| JA647 | CAACATGACTTAAAGTTCGGATGA | *cdc22*prom ChIP forward |
| JA648 | CCGGGAATATTTATAGTAAATGAAAAA | *cdc22* prom ChIP reverse |
| J1984 | AACCGTATGGGAGATTAGTCAAAAG | *cdc22* qPCR forward |
| J1985 | GATCCTTCAAGTGAATGTTCAAACT | *cdc22* qPCR reverse |
| J2010 | GAAACACCTTTTATACATCCTGCTG | *cdc18* qPCR forward |
| J2011 | GCATGCCTACATATGTCTAATGCTT | *cdc18* qPCR reverse |
| J2026 | TGGTAGAGCAAGTCACTGTTAATGA | 5’ ChIP mtDNA (control) |
| J2027 | CGTTATAACCGAGCTAACATCAAAC | 3’ ChIP mtDNA (control) |
| J2146 | ATGCTAGCTGTTGATGAAAGAATG | srw1 ChIP d forward |
| J2147 | TGTTTAGTGACATGTTACCCAGTTG | srw1 ChIPd reverse |
| J2148 | TGATTAACTTATCCAAAAACCAGTCC | srw1 ChIP p forward |
| J2149 | TCGACATAAATGGCTAGTAATTCAAA | srw1 ChIP p reverse |
| J3232 | gactGGTGAAGTAAAAATGATAGT | ubc11 sgRNA, forward |
| J3233 | aaacACTATCATTTTTACTTCACC | ubc11 sgRNA, reverse |
| J3234 | CGACTAACGTAACGAAAATAGTACTATG  AAGGATTGAAATTTAAAATTTCAATGTCA  TTTCCAGCTAATTACCCATATTCCCCTCT  AACTATCATTTTTA | ubc11-P93L CRISPR HR, forward |
| J3235 | GACTGCTGACCATTTATCTTTTAAAATGTC  TAAACATATGTTTCCACTCATATCGACATT  GGGGTGCCACATTGGTGAAGTAAAAATGA  TAGTTAGAGGG | ubc11-P93L CRISPR HR, reverse |
| J3236 | gactCATACCTTTGGTGGCTTGAA | ubc4 sgRNA, forward |
| J3237 | aaacTTCAAGCCACCAAAGGTATG | ubc4 sgRNA, reverse |
| J3238 | GCAGCTAACATGATATATAGGCTGACAGC  CCTTATGCGGGTGGTGTCTTCTTCTTGTCC  ATTCATTTCCCTACGGACTACTCATTCAAGC  CACCAAAGGT | ubc4-P61S CRISPR HR, forward |
| J3239 | GTATATAACAAACTTCCTCCTCCGATTCACA  AGCTTACAATGCGTATTTACATAATCTACAC  AAGACTAAATAGTTACATACCTTTGGTGGCT  TGAATGA | ubc4-P61S CRISPR HR, reverse |
| OLEH842 | CTGTTCAGGTTTTGCACTTTTTATT | *tfb2* qPCR forward |
| OLEH843 | TTCAAGCATGATTTGTTGTGTATCT | *tfb2* qPCR reverse |
