# Supplementary material for: Nrm1 is a bistable switch connecting cell cycle progression to transcriptional control
Source: EMBO Rep. 2025 Aug 29;26(20):5048–69. doi: 10.1038/s44319-025-00566-7 (PMC12550009; doi:10.1038/s44319-025-00566-7)
Supplement: Supplementary file 4 — Appendix [file 44319_2025_566_MOESM4_ESM.pdf]

Appendix

Nrm1 is a bistable switch connecting cell cycle progression to transcriptional control

Guillem Murciano-Julià, Montserrat Vega, Esther Pazo, Àlex Pascual-Serra, Isabel Alves-Rodrigues, Oriol Bagudanch, Roger Anglada, Núria Bonet, Rosa Aligué, Sergio Moreno, Baldo Oliva, Elena Hidalgo and José Ayté

Table of Content

Appendix Figure S1……………………………………..2

Appendix Figure S2……………………………………..3

Appendix Figure S3……………………………………..4

Appendix Figure S4……………………………………..5

Appendix Figure S5……………………………………..6

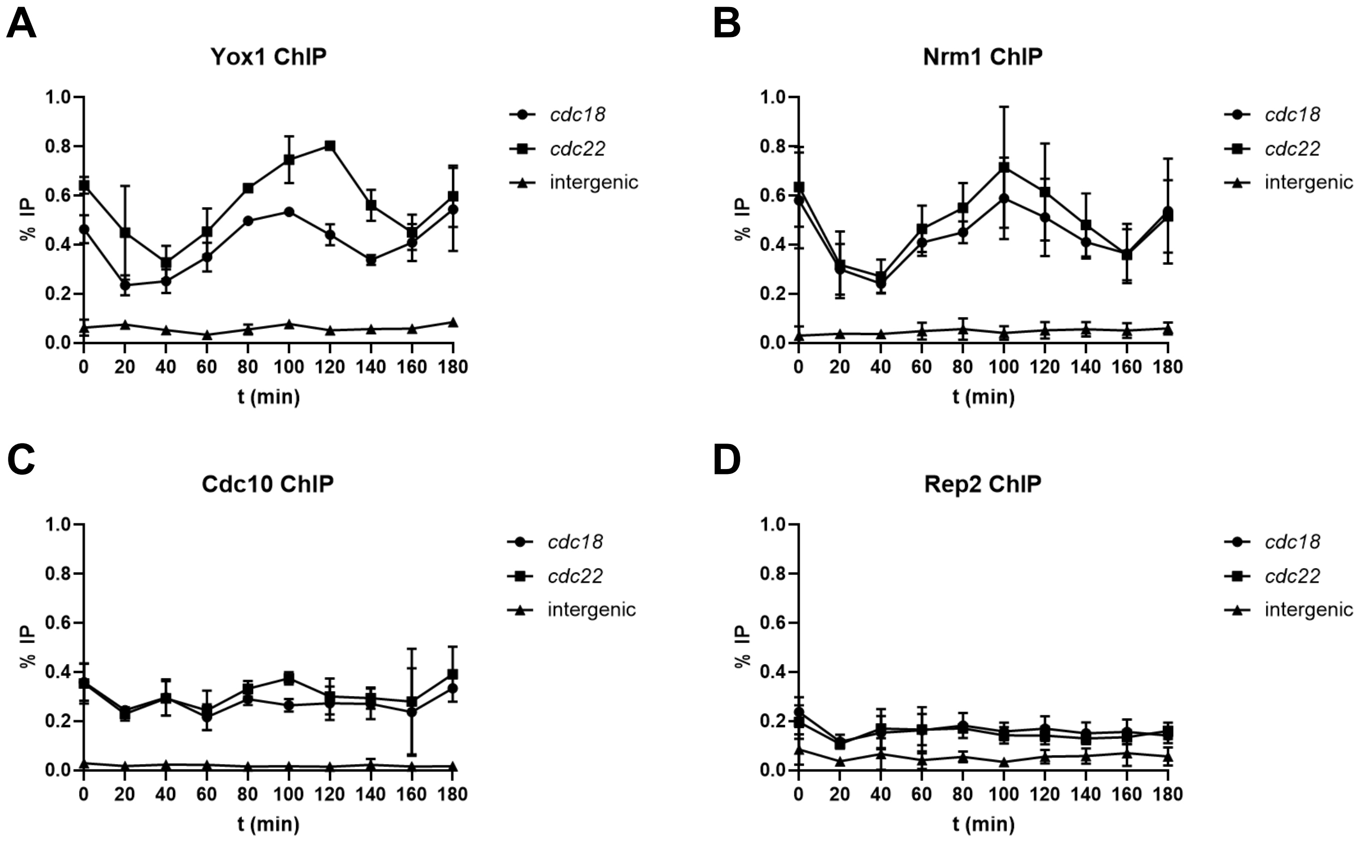

Appendix Figure S1.

ChIP experiments from Fig. 2A showing each immunoprecipitated protein in individual graphs, with interaction to *cdc18* and *cdc22* promoters and an intergenic region as control. (**A**) Yox1 ChIP; (**B**) Nrm1-HA ChIP; (**C**) Cdc10 ChIP; (**D**) Rep2-TAP ChIP.

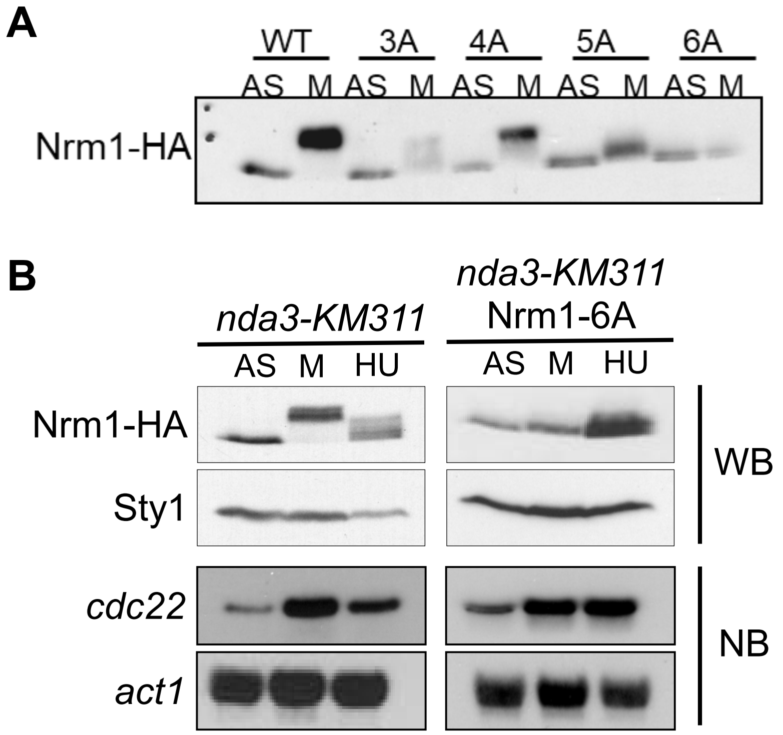

Appendix Figure S2.

(**A**) Western blot analysis of asynchronous (AS) or metaphase arrested (M) *nda3-KM311* cells with wild type Nrm1 (WT) or different phosphorylation mutants: 3A (T9A, S57A, T287A), 4A (T9A, S57A, T116A, T287A); 5A (T9A, S57A, S237A, T241A, T287A); 6A (T9A, S57A, T116A, S237A, T241A, T287A). Cells were grown in YE5S at 30ºC and shifted at 18ºC for 5h for metaphase arrest prior to TCA sample collection.

(**B**) Western Blot (WB) and Northern Blot from *nda3-KM311* strain expressing Nrm1-HA (left) or Nrm1-6A-HA (right); asynchronous culture (AS), cells arrested in metaphase for 5 hours at 18ºC (M), or cells treated with 10 mM Hydroxyurea (HU) for 3 hours at 30ºC. Sty1 is shown as loading control in the Western Blot and act1as a control in the Northern blot. Representative experiments of at least three different experiments are shown.

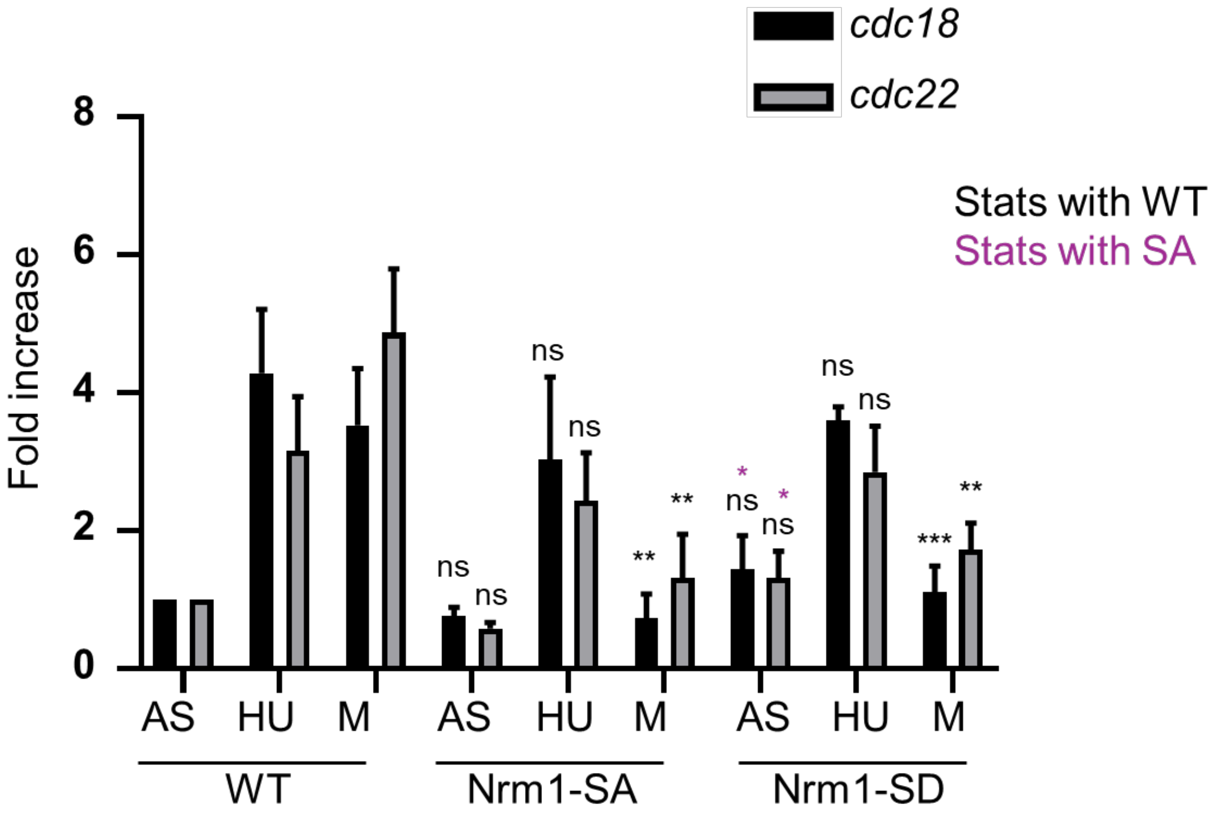

Appendix Figure S3.

qPCR transcription analysis of *cdc18* and *cdc22* expression in the Nrm1-HA, Nrm1-SA-HA and Nrm1-SD-HA in the *nda3-KM311* background. Cells were grown and treated as in Fig. 2B. AS, asynchronous culture; HU, cells treated with 10 mM Hydroxyurea for 3 hours at 30ºC; M; cells arrested in metaphase for 5 hours at 18ºC. *tfb2* was used as control gene. Plot represents mean ± SD of at least n=3 experiments. Statistics show significance from a student's T-test. In black relative to the same treatment with WT and in purple to the same treatment with SA. *: p<0.05; **: p<0.01; ***: p<0.001

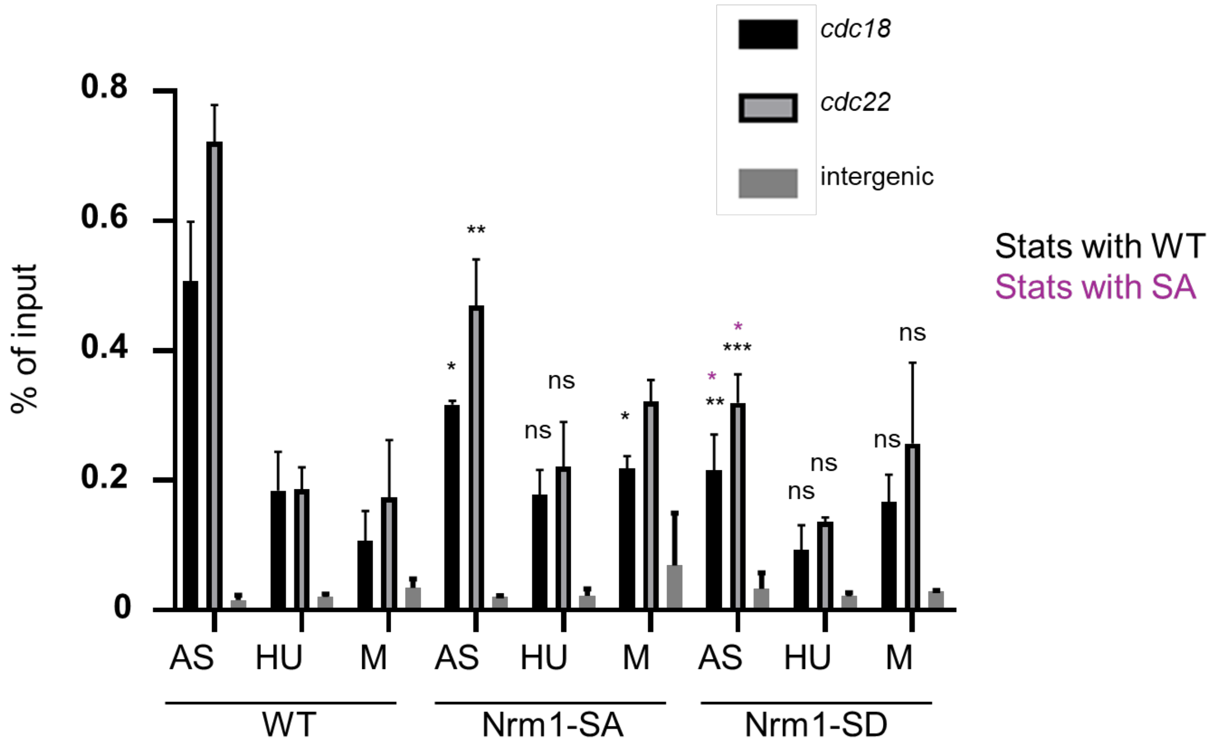

Appendix Figure S4.

ChIP analysis of Nrm1-HA, Nrm1-SA-HA and Nrm1-SD-HA in *nda3-KM311* strain. ChIP was done as described with α-HA antibodies. The isolated DNA was used to amplify the promoter region of *cdc18* or *cdc22*. Cells were grown and treated as in Fig. 2B. AS, asynchronous culture; HU, cells treated with 10 mM Hydroxyurea for 3 hours at 30ºC; M, cells arrested in metaphase for 5 hours at 18ºC. Plot represents mean ± SD of at least n=3 experiments. In black relative to the same treatment with WT and in purple to the same treatment with SA. Statistics show significance from a student's T-test. *: $p<0.05$; **: $p<0.01$; ***: $p<0.001$

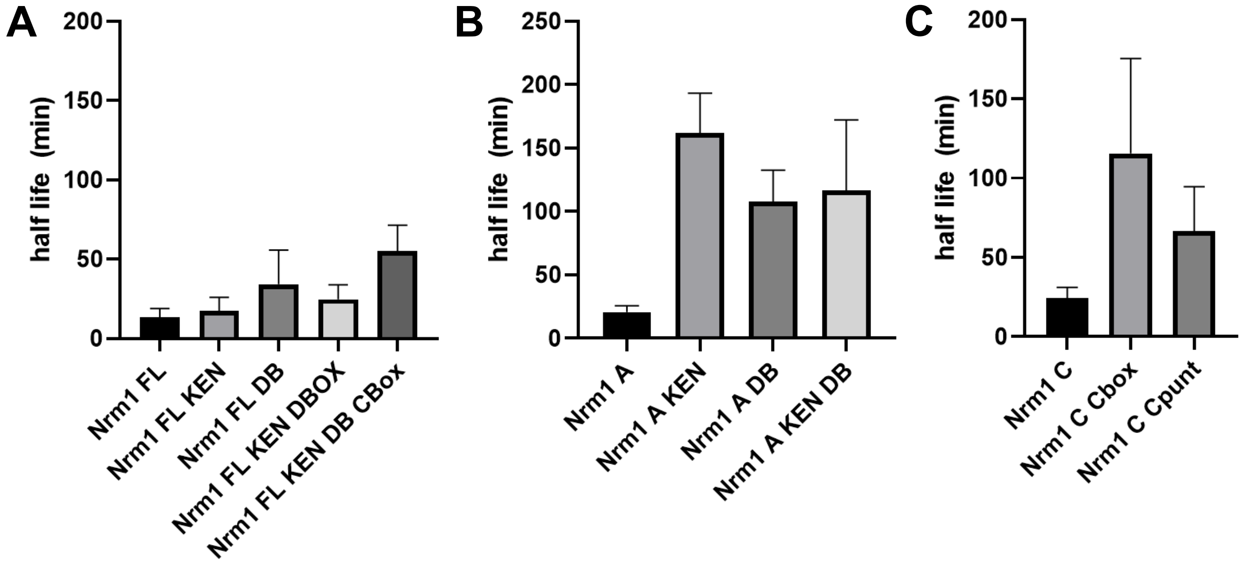

Appendix Figure S5.

(**A**) Half-life in minutes of Nrm1 full length (FL), Nrm1 FL ΔKEN, Nrm1 FL ΔDB, Nrm1 FL ΔKEN ΔDB and Nrm1 FL ΔKEN ΔDB ΔCBox after thiamine and cycloheximide (CHX) addition. TCA samples were collected at 0, 10, 30 and 60 minutes after CHX addition and analyzed in Western blots. Sample loading was normalized with α-Sty1. Half-life was calculated using GraphPad Prism. Plot represents mean ± SD of at least n = 3 experiments.

(**B**) Half-life in minutes of Nrm1 A, Nrm1 A ΔKEN, Nrm1 A ΔDB and Nrm1 A ΔKEN ΔDB after thiamine and cycloheximide (CHX) addition. TCA samples were collected at 0, 10, 30 and 60 minutes after CHX addition and analyzed in Western blots. Sample loading was normalized with α-Sty1. Half-life was calculated using GraphPad Prism. Plot represents mean ± SD of at least n = 3 experiments.

(**C**) Half-life in minutes of Nrm1 C, Nrm1 C ΔCBox and Nrm1 C ΔCpunt after thiamine and cycloheximide (CHX) addition. TCA samples were collected at 0, 10, 30 and 60 minutes after CHX addition and analyzed in Western blots. Sample loading was normalized with α-Sty1. Half-life was calculated using GraphPad Prism. Plot represents mean ± SD of at least n = 3 experiments.
