# Supplementary figures and images for: Nrm1 is a bistable switch connecting cell cycle progression to transcriptional control
Source: EMBO Rep. 2025 Aug 29;26(20):5048–69. doi: 10.1038/s44319-025-00566-7 (PMC12550009; doi:10.1038/s44319-025-00566-7)

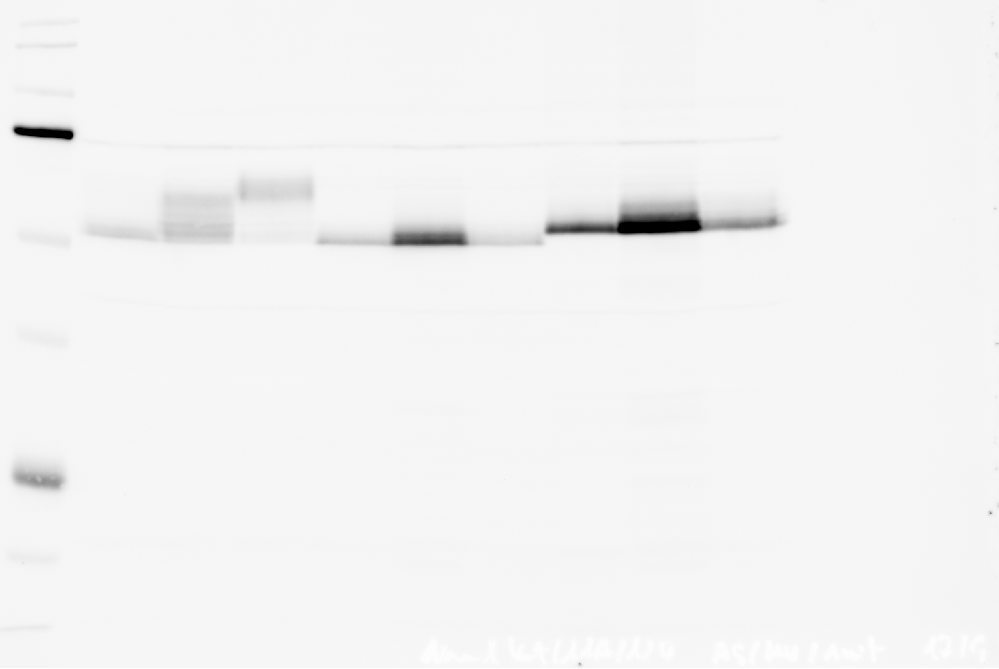

Supplement: Supplementary file 8 — Source data Fig. 3 [file 44319_2025_566_MOESM8_ESM.zip › Fig 3/3B/3B HA.tif]

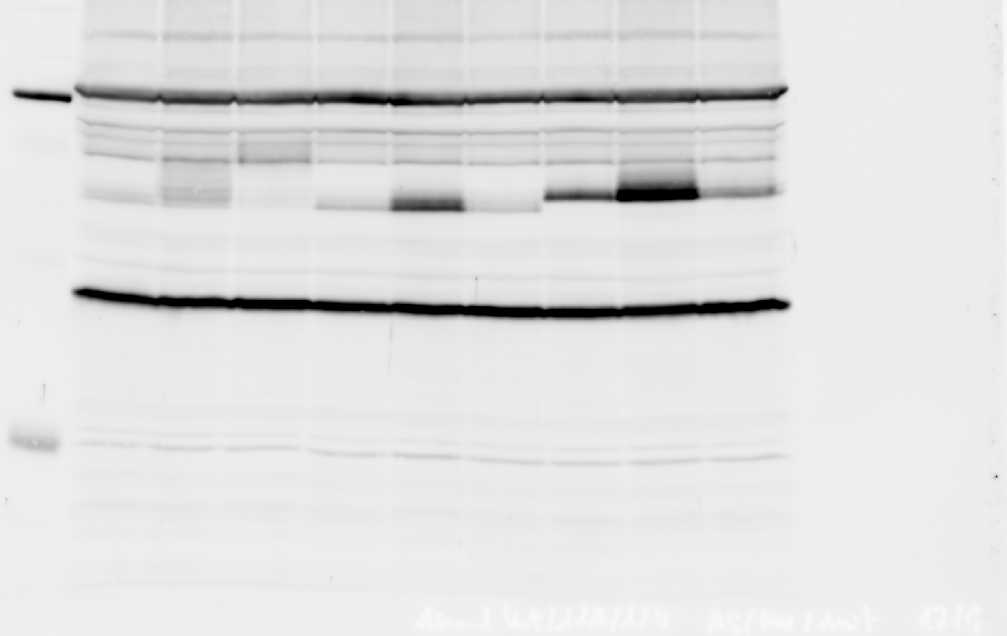

Supplement: Supplementary file 8 — Source data Fig. 3 [file 44319_2025_566_MOESM8_ESM.zip › Fig 3/3B/3B Sty1.tif]

## Slide 1
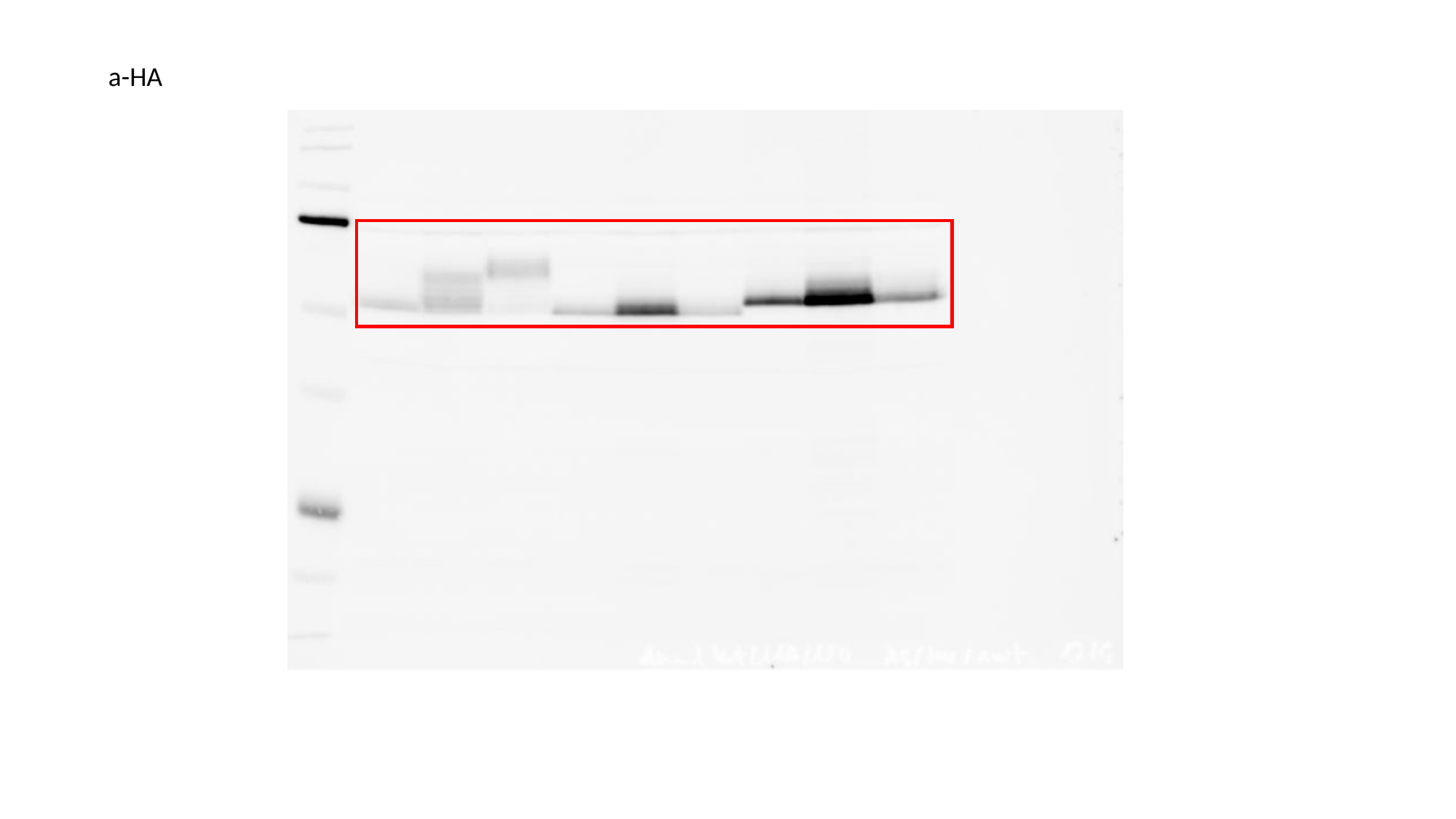

a-HA

## Slide 2
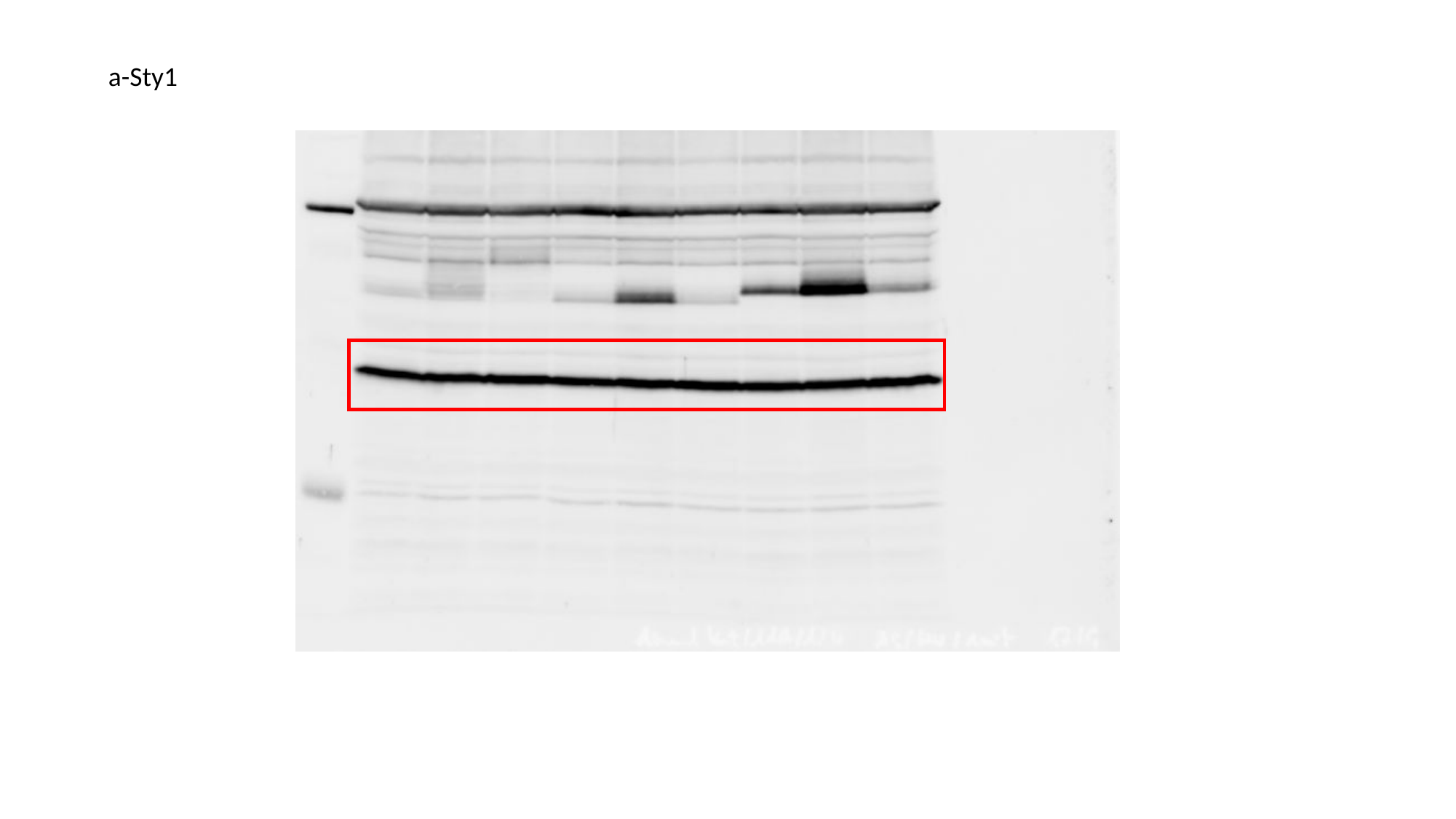

a-Sty1

Supplement: Supplementary file 8 — Source data Fig. 3 [file 44319_2025_566_MOESM8_ESM.zip › Fig 3/3B/3B WB CROPPINGS.pptx]

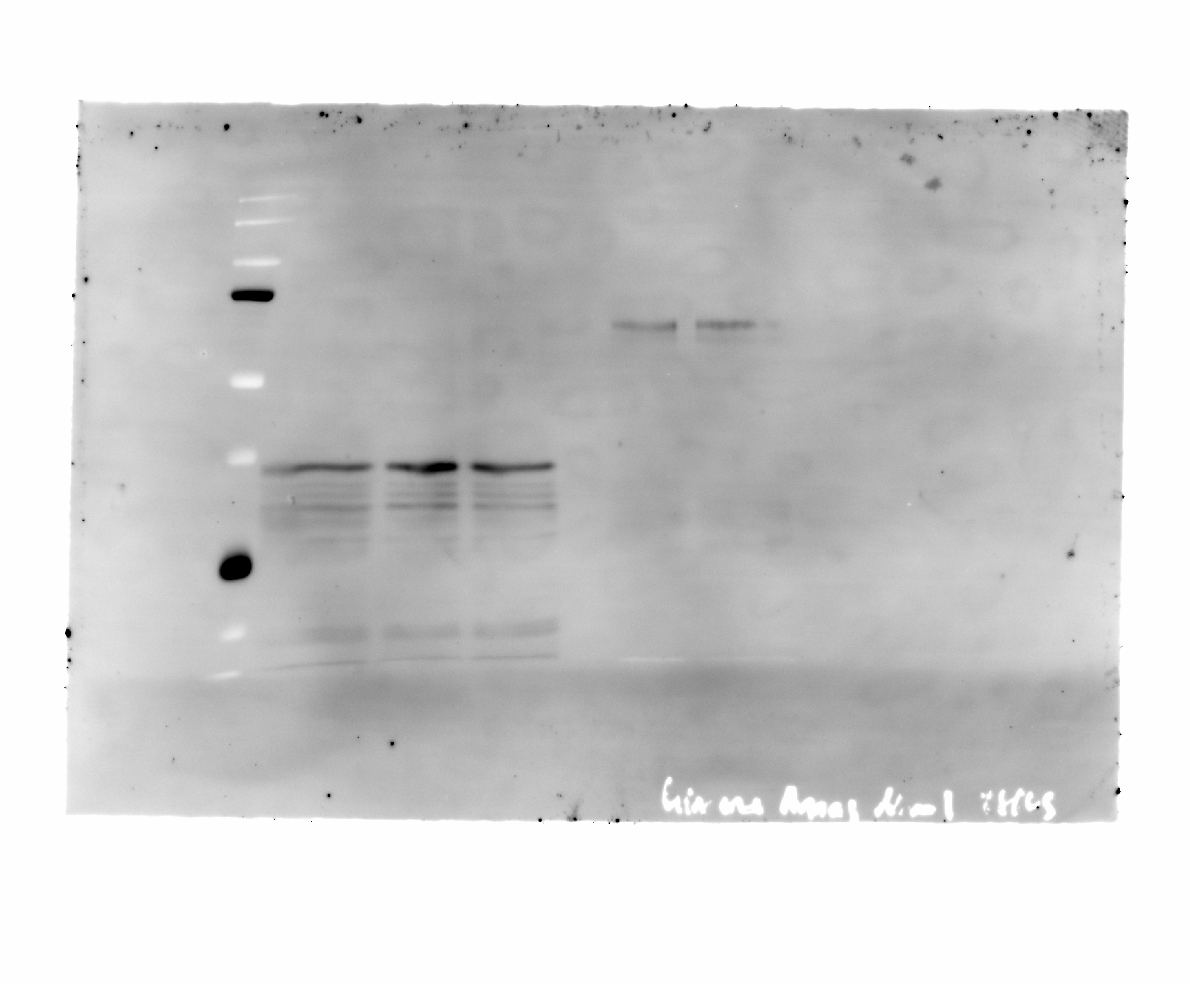

Supplement: Supplementary file 8 — Source data Fig. 3 [file 44319_2025_566_MOESM8_ESM.zip › Fig 3/3C/3C a-HA.tif]

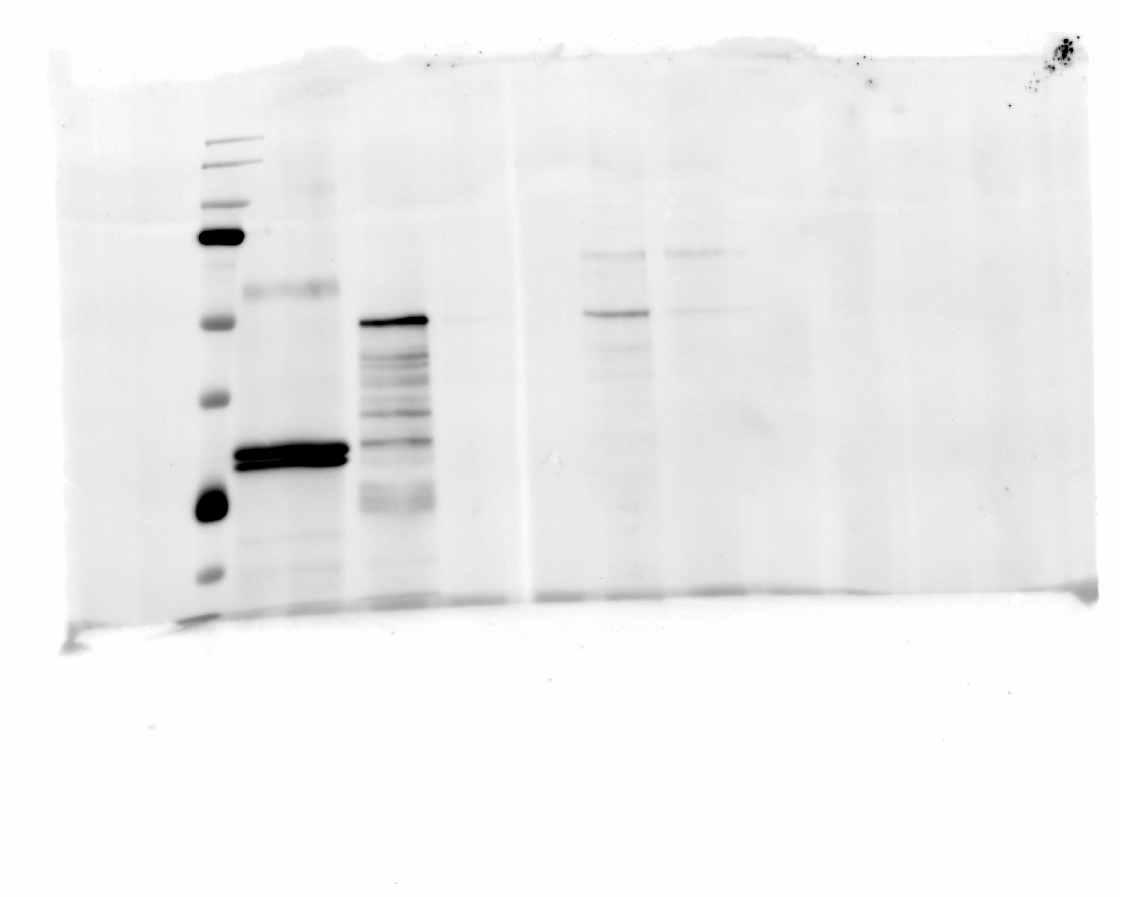

Supplement: Supplementary file 8 — Source data Fig. 3 [file 44319_2025_566_MOESM8_ESM.zip › Fig 3/3C/3C a-thiophosphate.tif]

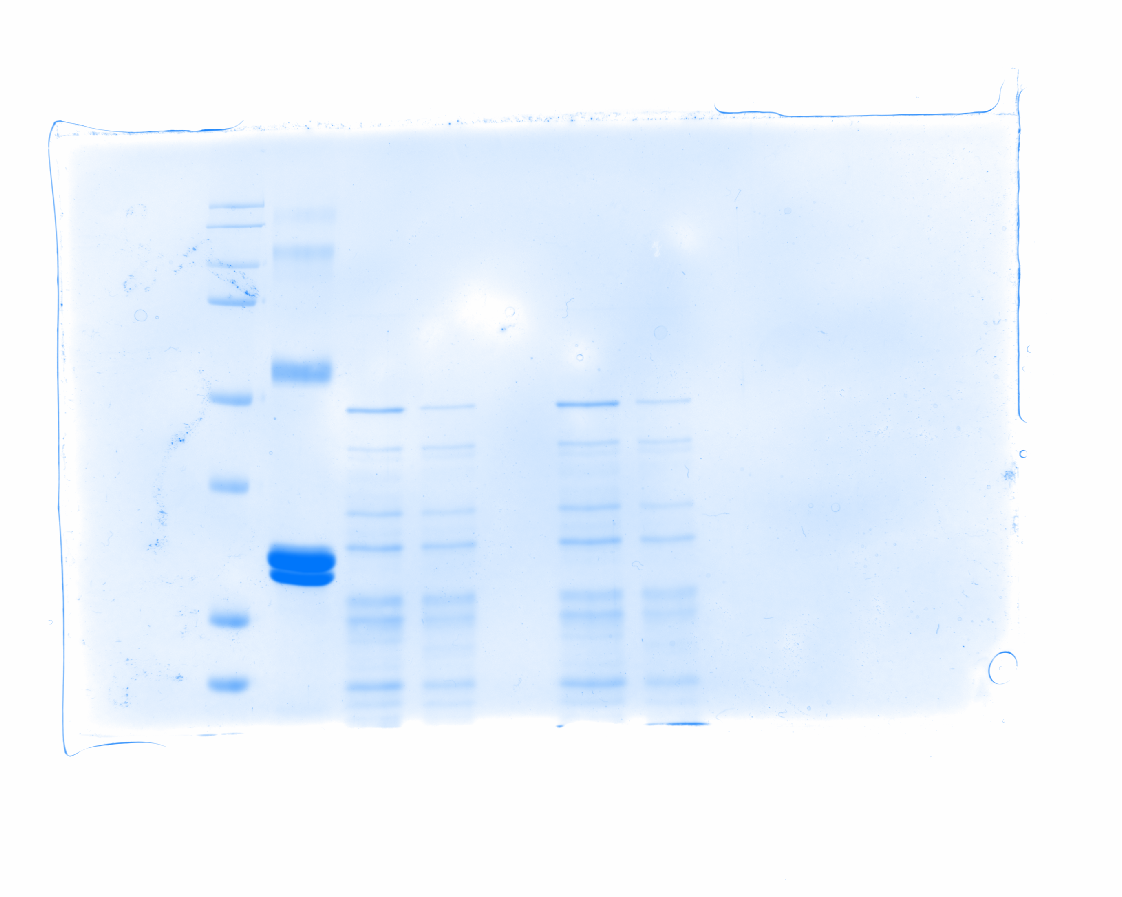

Supplement: Supplementary file 8 — Source data Fig. 3 [file 44319_2025_566_MOESM8_ESM.zip › Fig 3/3C/3C coomassie.tif]

## Slide 1
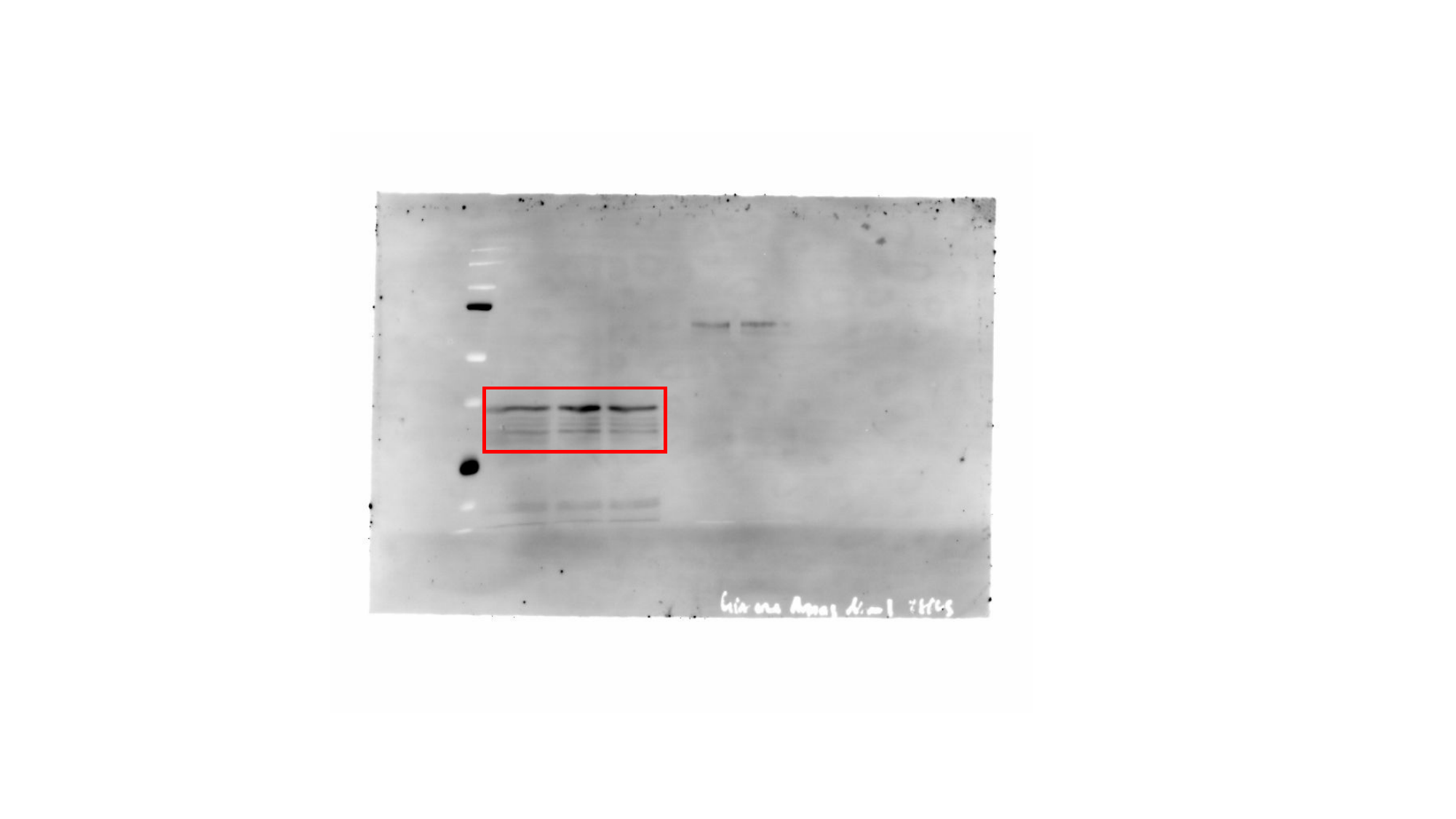

## Slide 2
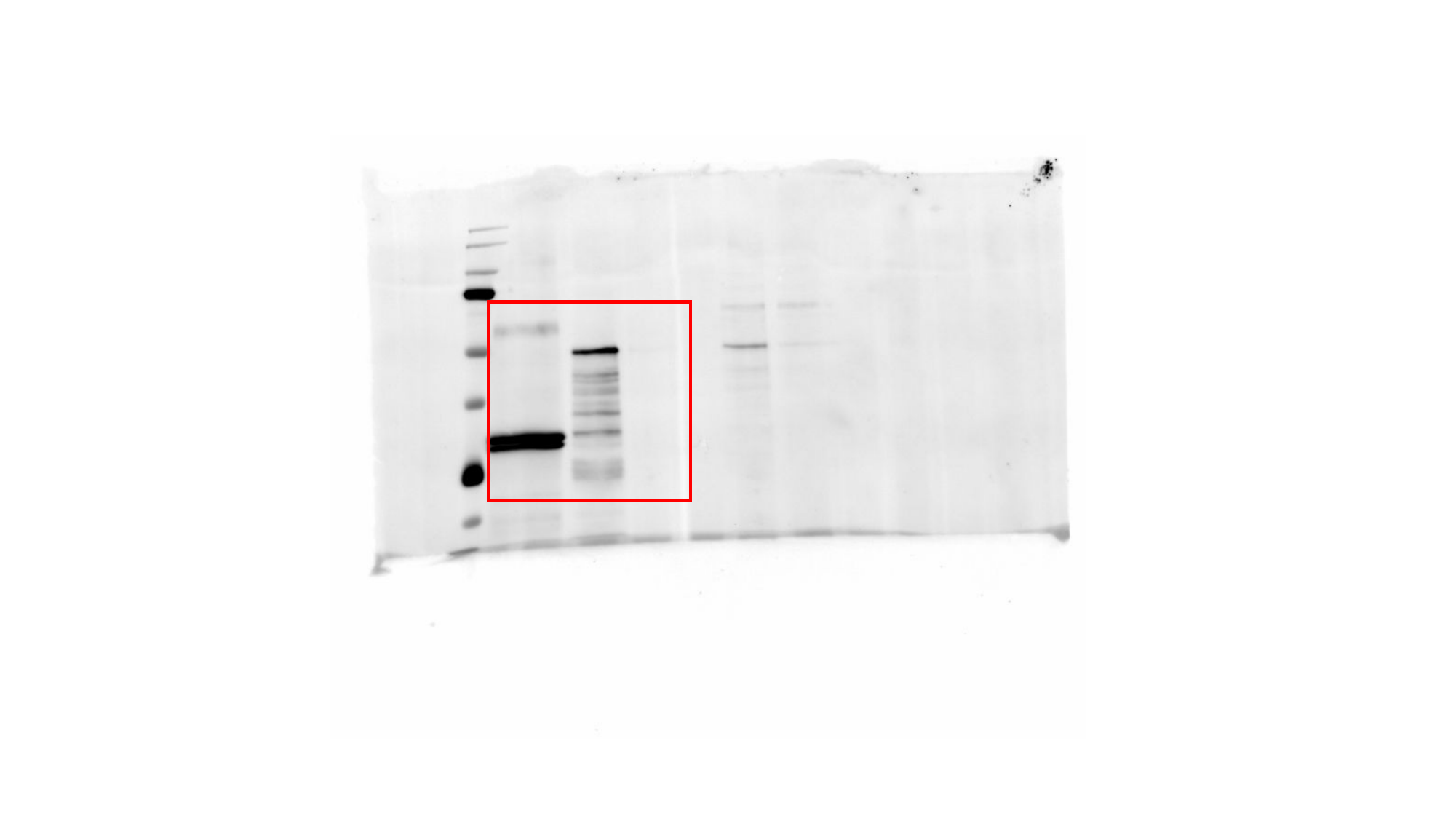

## Slide 3
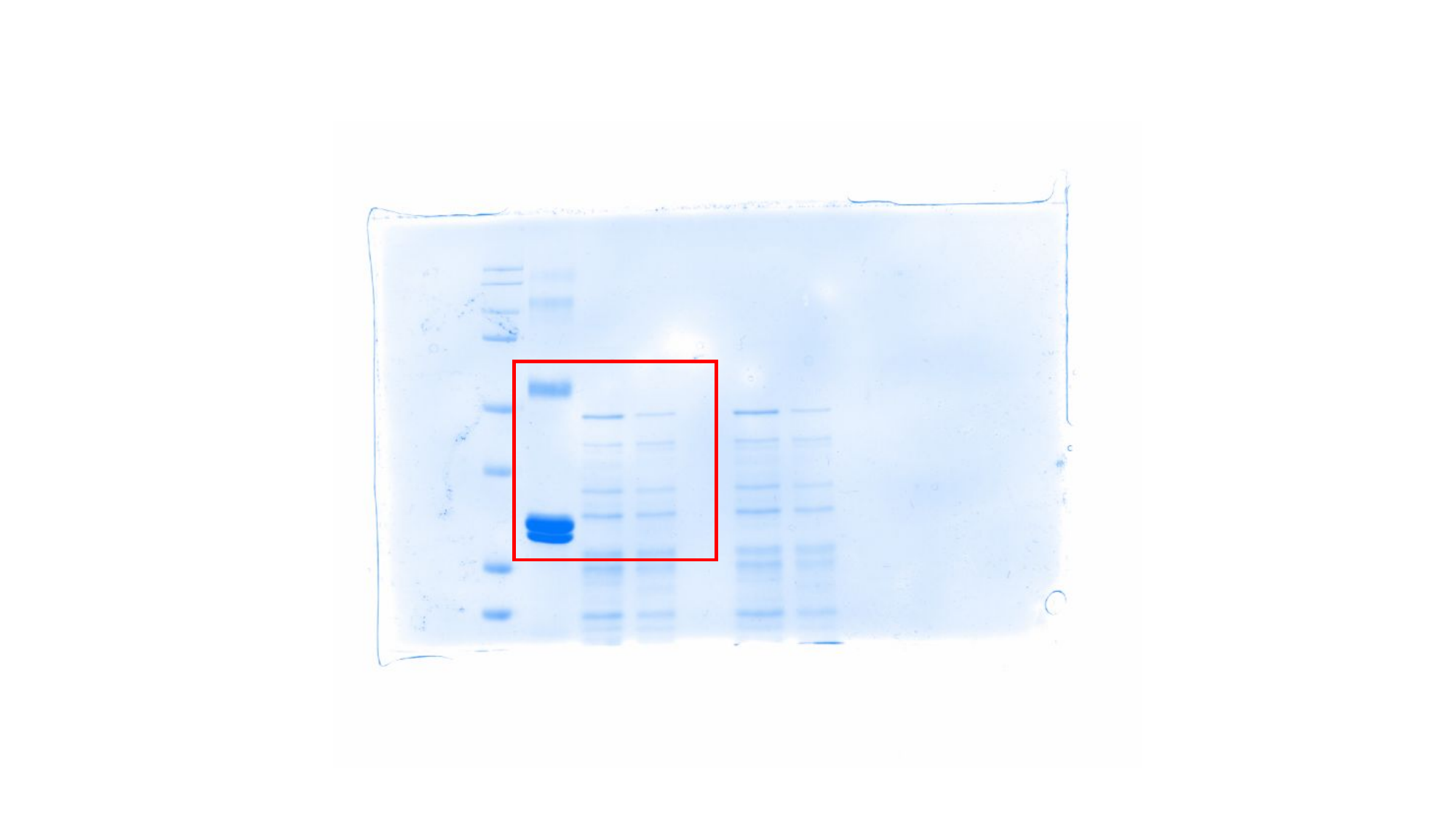

Supplement: Supplementary file 8 — Source data Fig. 3 [file 44319_2025_566_MOESM8_ESM.zip › Fig 3/3C/3D WB CROPPINGS.pptx]

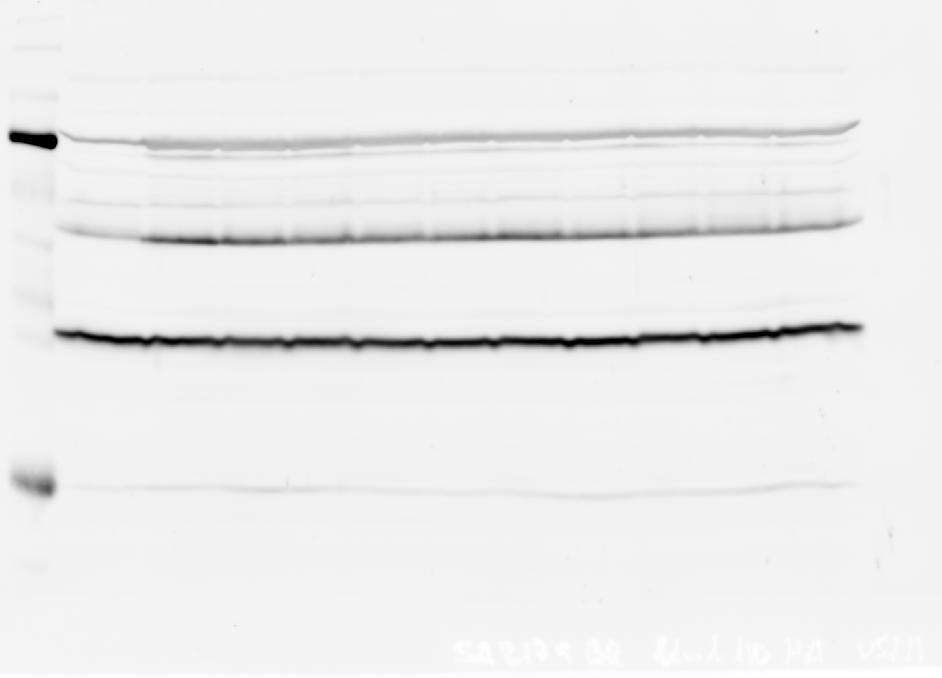

Supplement: Supplementary file 9 — Source data Fig. 4 [file 44319_2025_566_MOESM9_ESM.zip › Fig 4/4A/4A - Nrm-SD a-Sty1.tif]

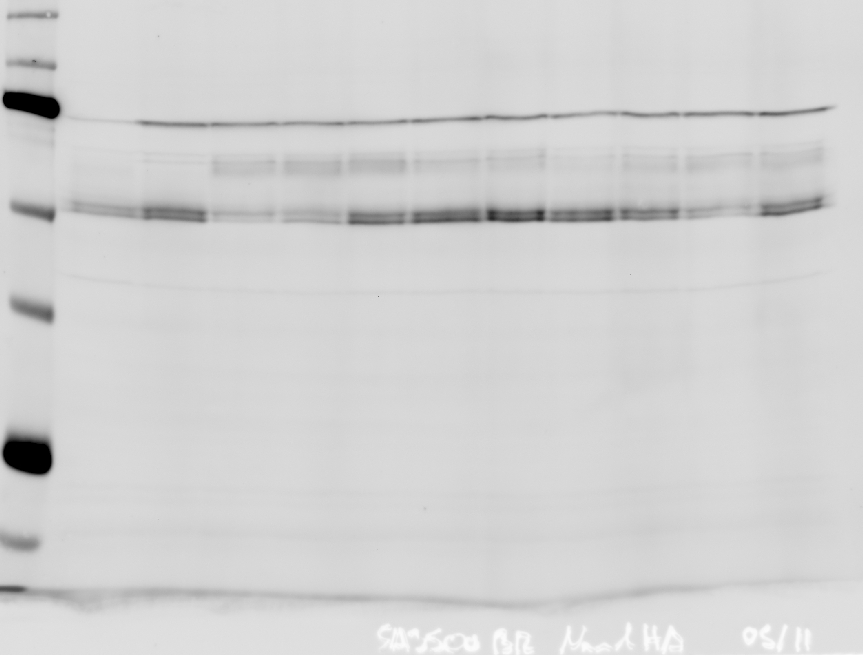

Supplement: Supplementary file 9 — Source data Fig. 4 [file 44319_2025_566_MOESM9_ESM.zip › Fig 4/4A/4A - Nrm1-HA a-HA.tif]

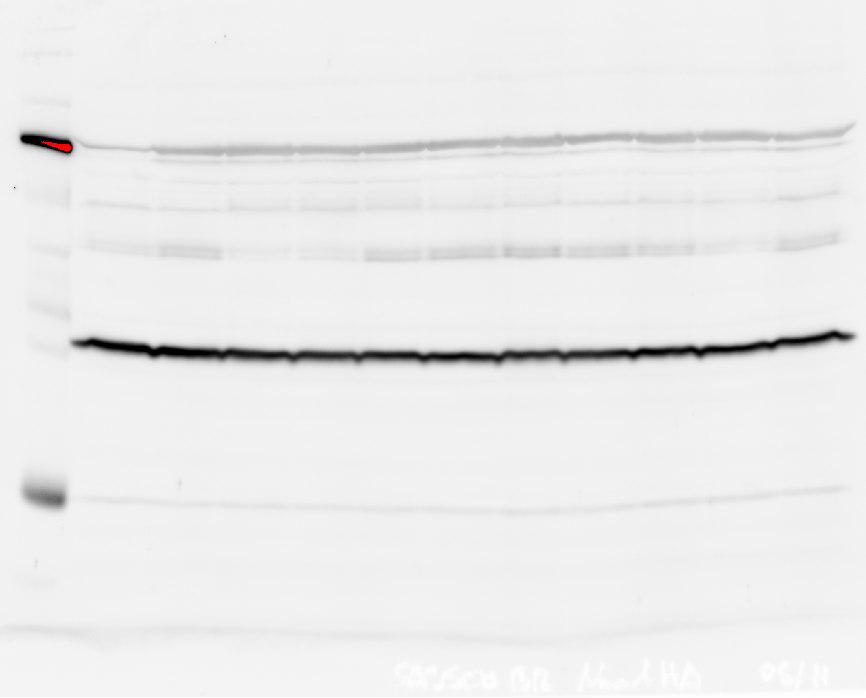

Supplement: Supplementary file 9 — Source data Fig. 4 [file 44319_2025_566_MOESM9_ESM.zip › Fig 4/4A/4A - Nrm1-HA a-Sty1.tif]

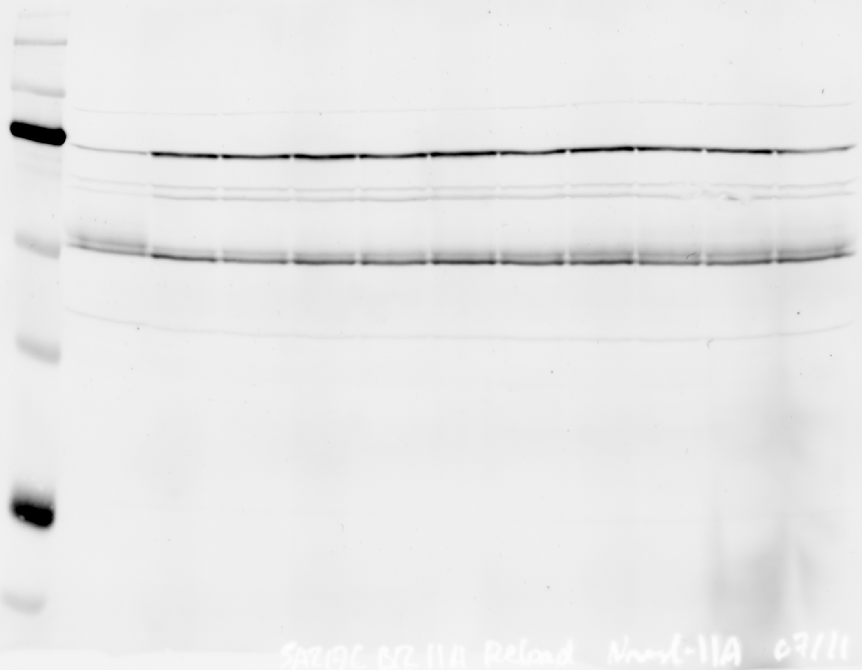

Supplement: Supplementary file 9 — Source data Fig. 4 [file 44319_2025_566_MOESM9_ESM.zip › Fig 4/4A/4A - Nrm1-SA a-HA.tif]

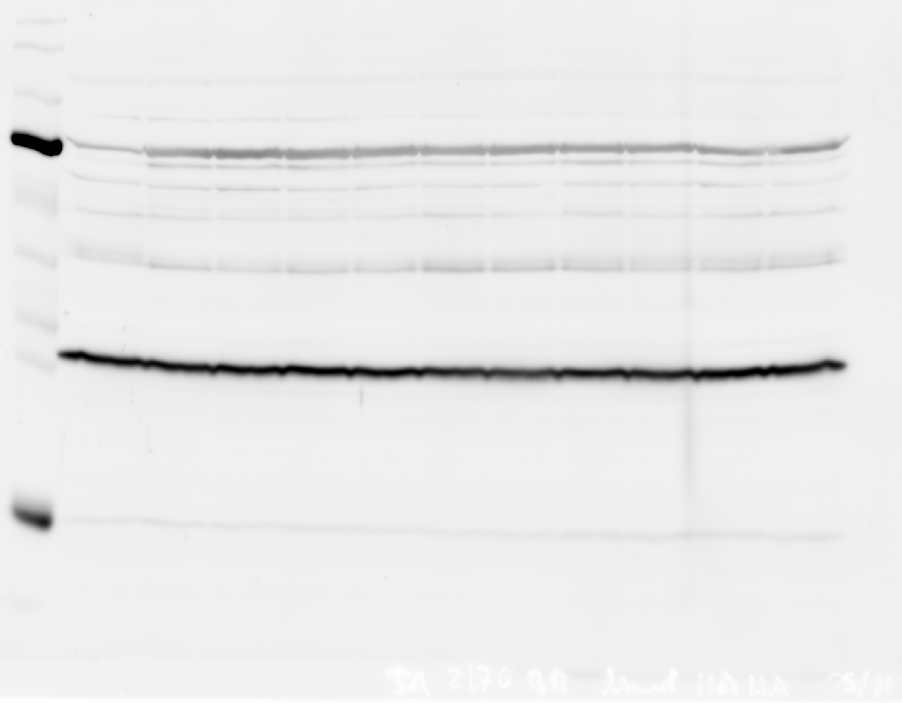

Supplement: Supplementary file 9 — Source data Fig. 4 [file 44319_2025_566_MOESM9_ESM.zip › Fig 4/4A/4A - Nrm1-SA a-Sty1.tif]

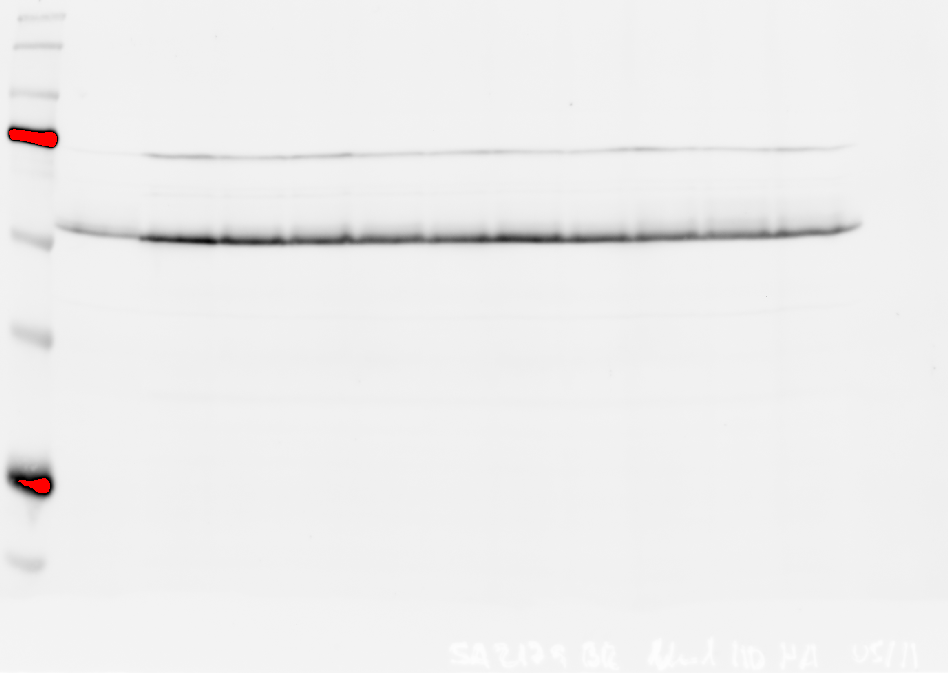

Supplement: Supplementary file 9 — Source data Fig. 4 [file 44319_2025_566_MOESM9_ESM.zip › Fig 4/4A/4A - Nrm1-SD a-HA.tif]

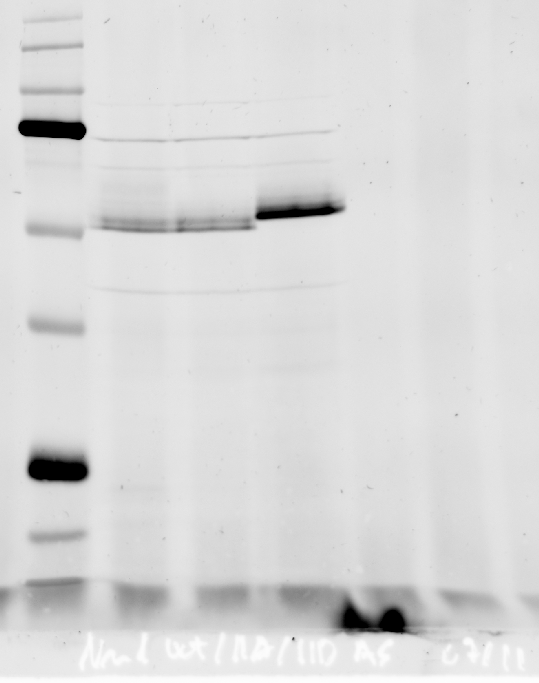

Supplement: Supplementary file 10 — Source data Fig. 5 [file 44319_2025_566_MOESM10_ESM.zip › Fig 5/5A/5A a-HA.tif]

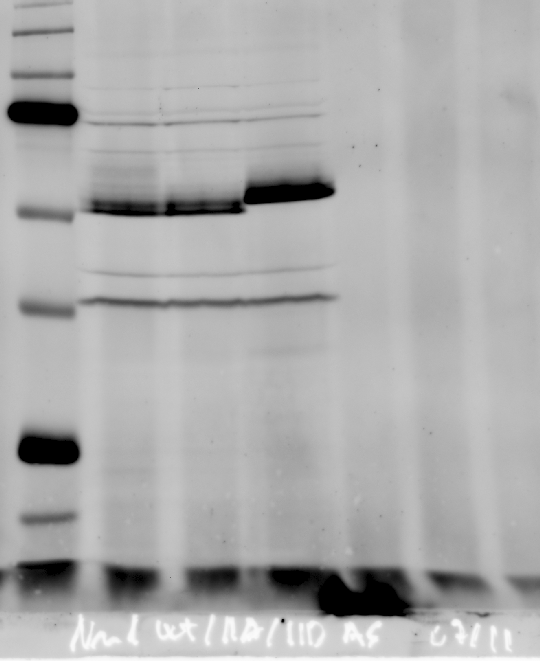

Supplement: Supplementary file 10 — Source data Fig. 5 [file 44319_2025_566_MOESM10_ESM.zip › Fig 5/5A/5A a-Sty1.tif]

## Slide 1
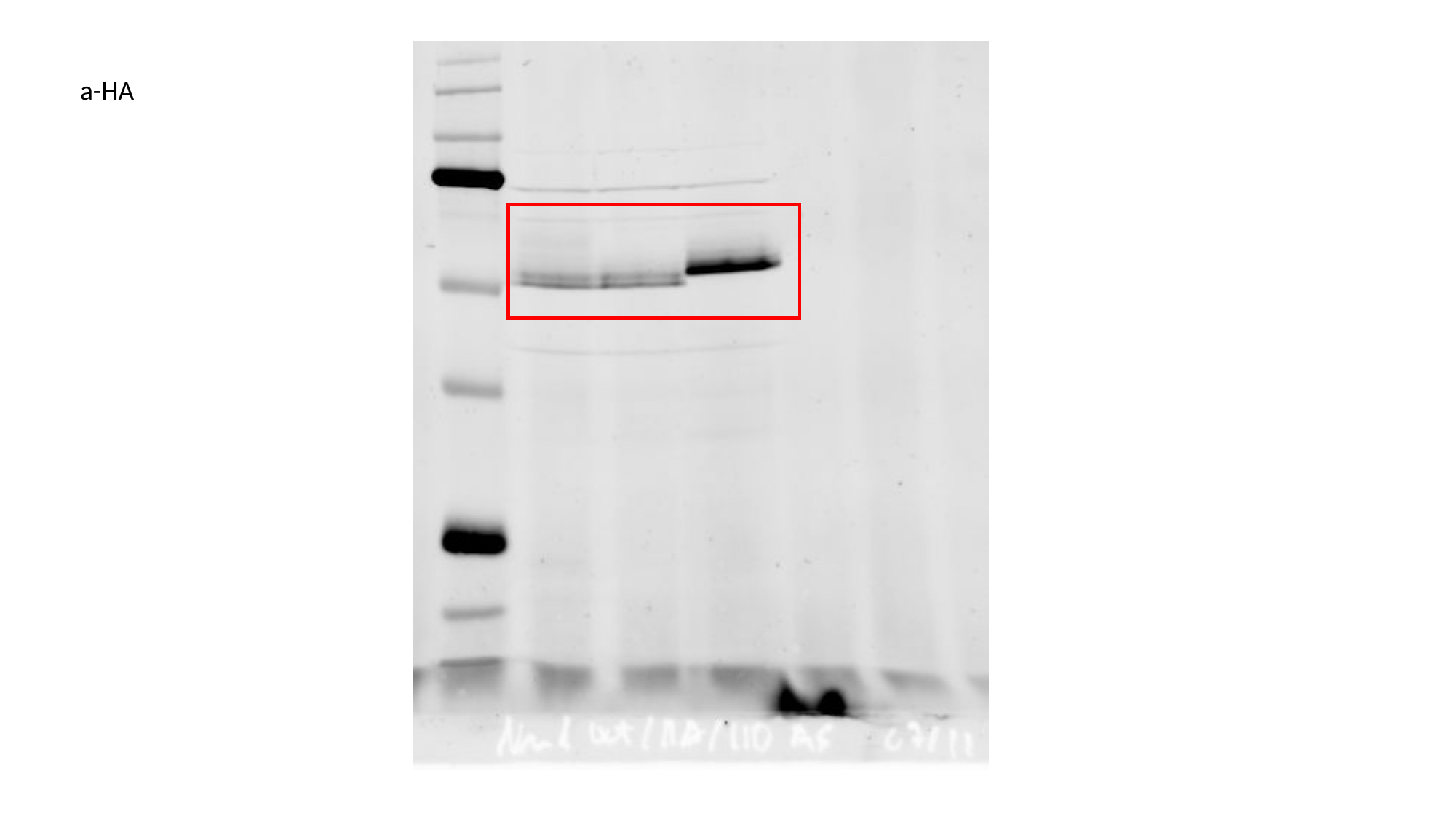

a-HA

## Slide 2
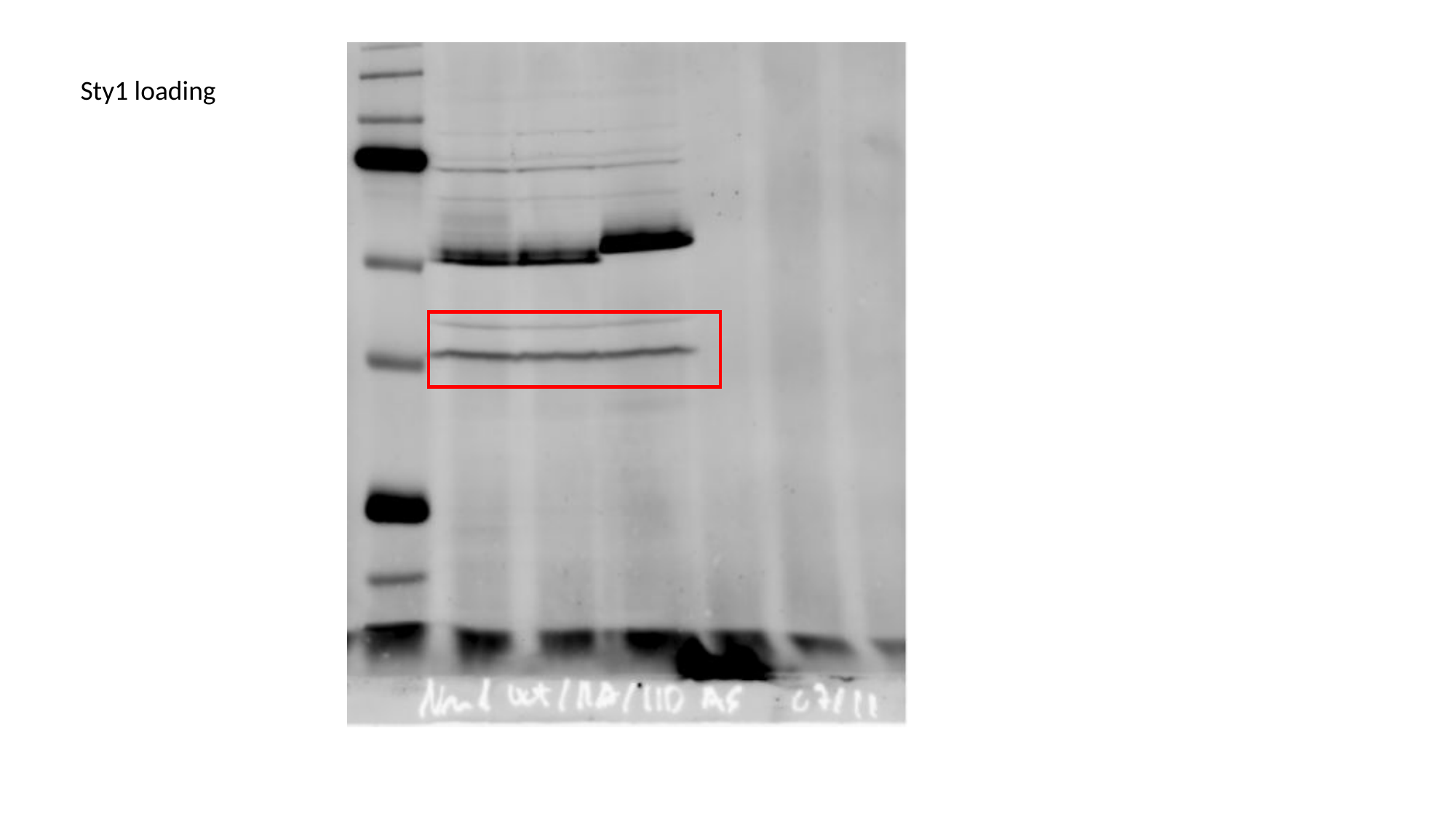

Sty1 loading

Supplement: Supplementary file 10 — Source data Fig. 5 [file 44319_2025_566_MOESM10_ESM.zip › Fig 5/5A/5A WB CROPPING.pptx]

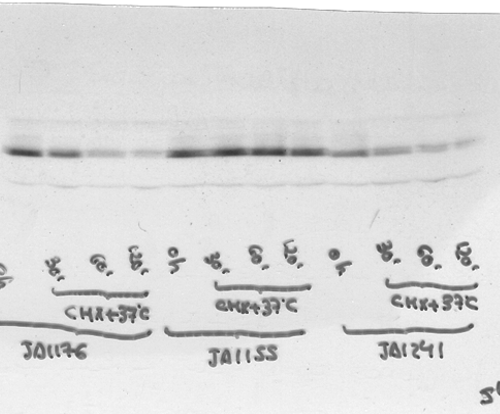

Supplement: Supplementary file 10 — Source data Fig. 5 [file 44319_2025_566_MOESM10_ESM.zip › Fig 5/5C/5C a-HA.tif]

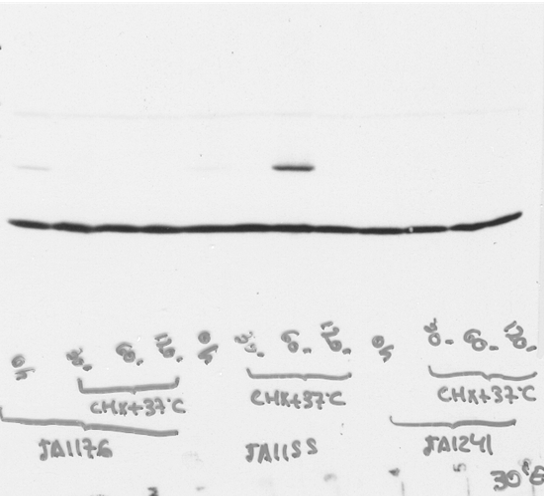

Supplement: Supplementary file 10 — Source data Fig. 5 [file 44319_2025_566_MOESM10_ESM.zip › Fig 5/5C/5C a-Sty1.tif]

## Slide 1
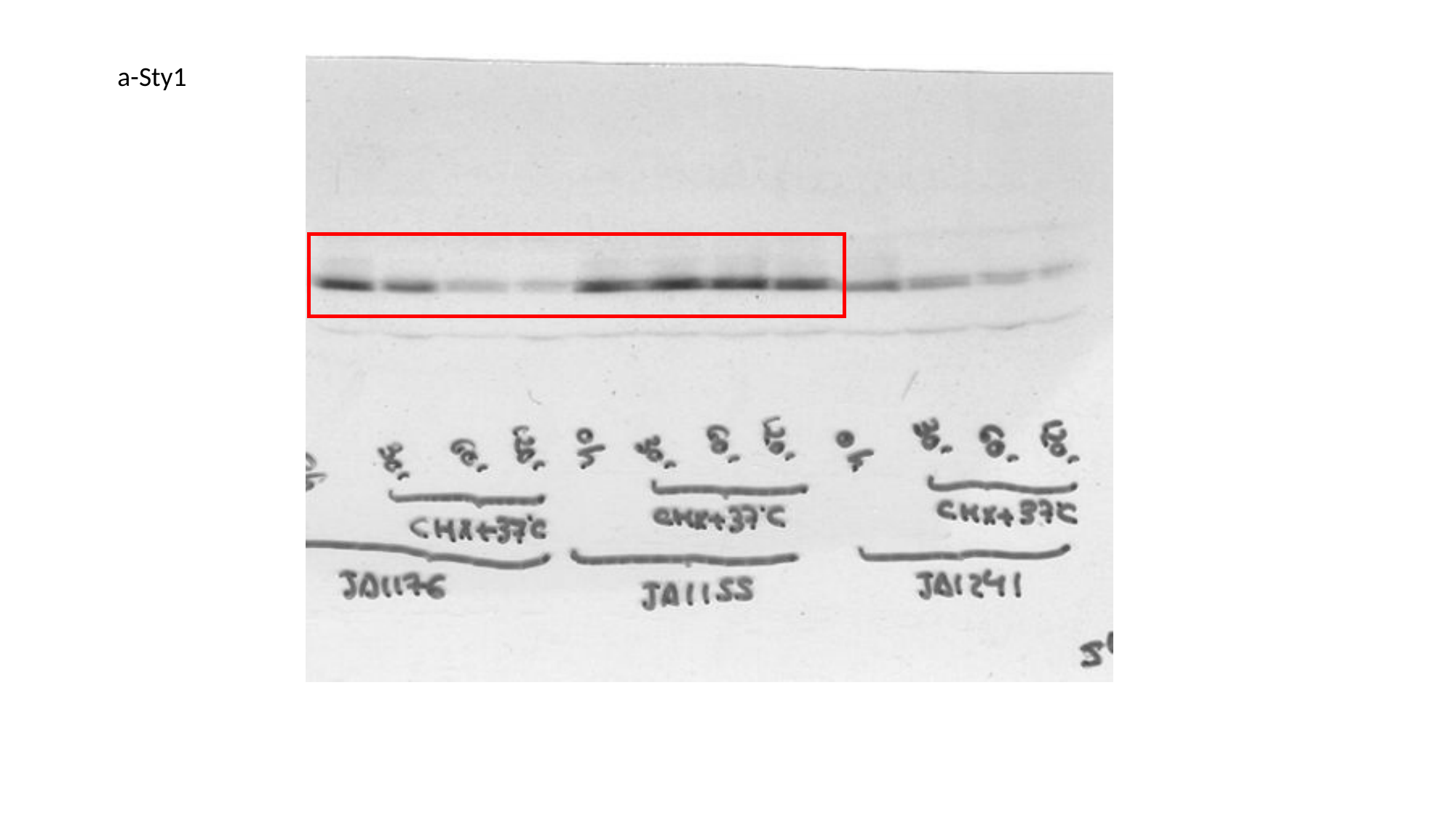

a-Sty1

## Slide 2
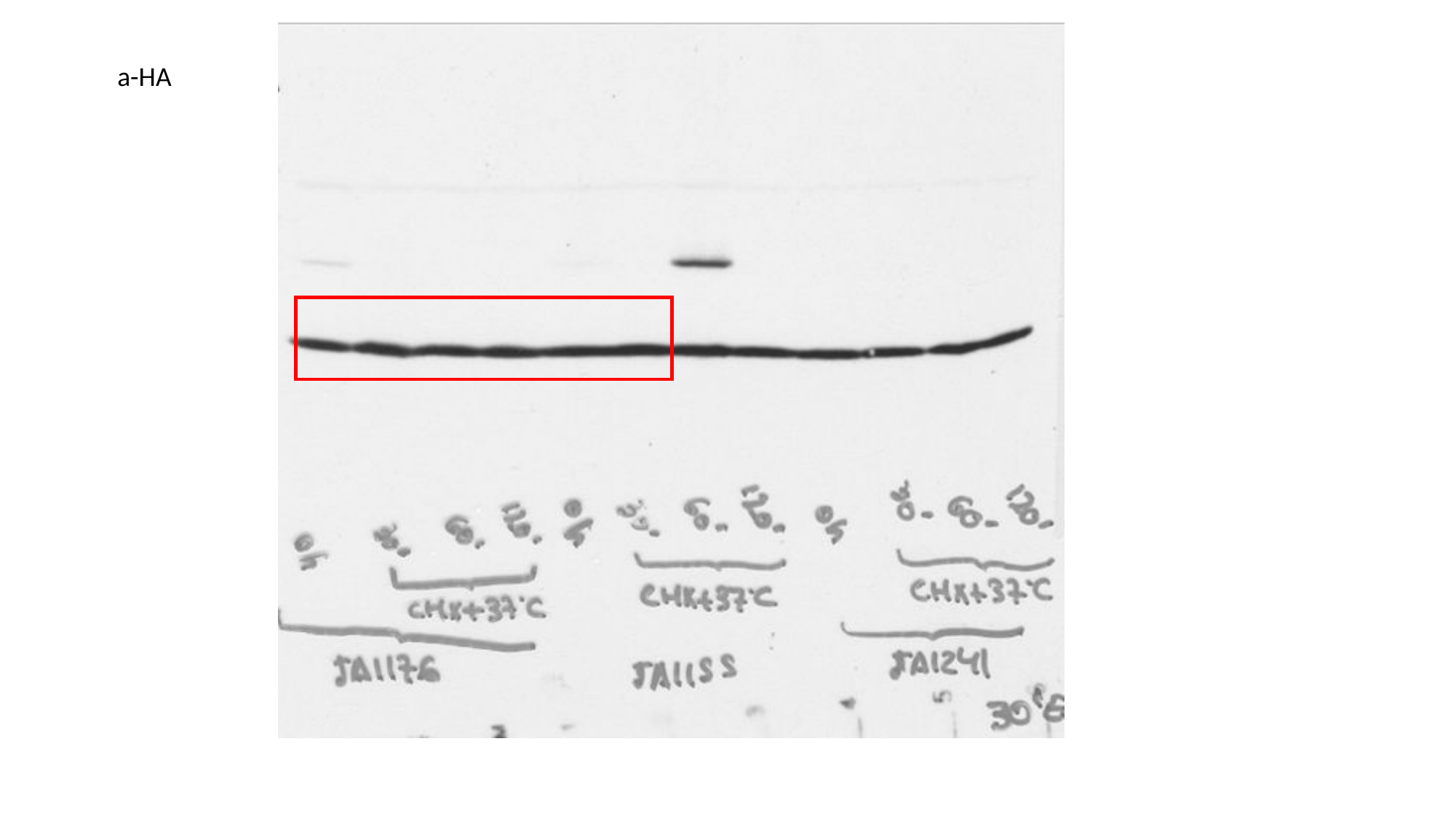

a-HA

Supplement: Supplementary file 10 — Source data Fig. 5 [file 44319_2025_566_MOESM10_ESM.zip › Fig 5/5C/5C WB CROPPINGS.pptx]

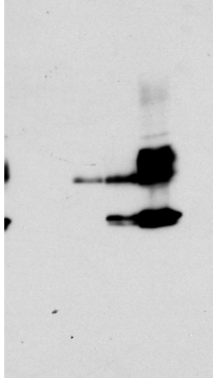

Supplement: Supplementary file 10 — Source data Fig. 5 [file 44319_2025_566_MOESM10_ESM.zip › Fig 5/5D/5D input.tif]

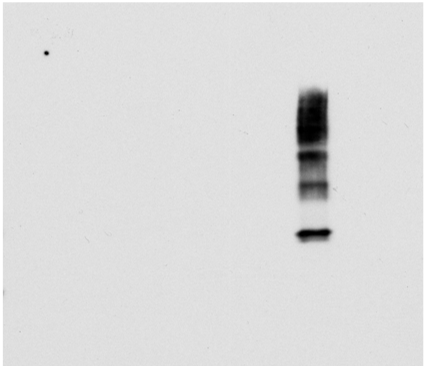

Supplement: Supplementary file 10 — Source data Fig. 5 [file 44319_2025_566_MOESM10_ESM.zip › Fig 5/5D/5D IP.tif]

## Slide 1
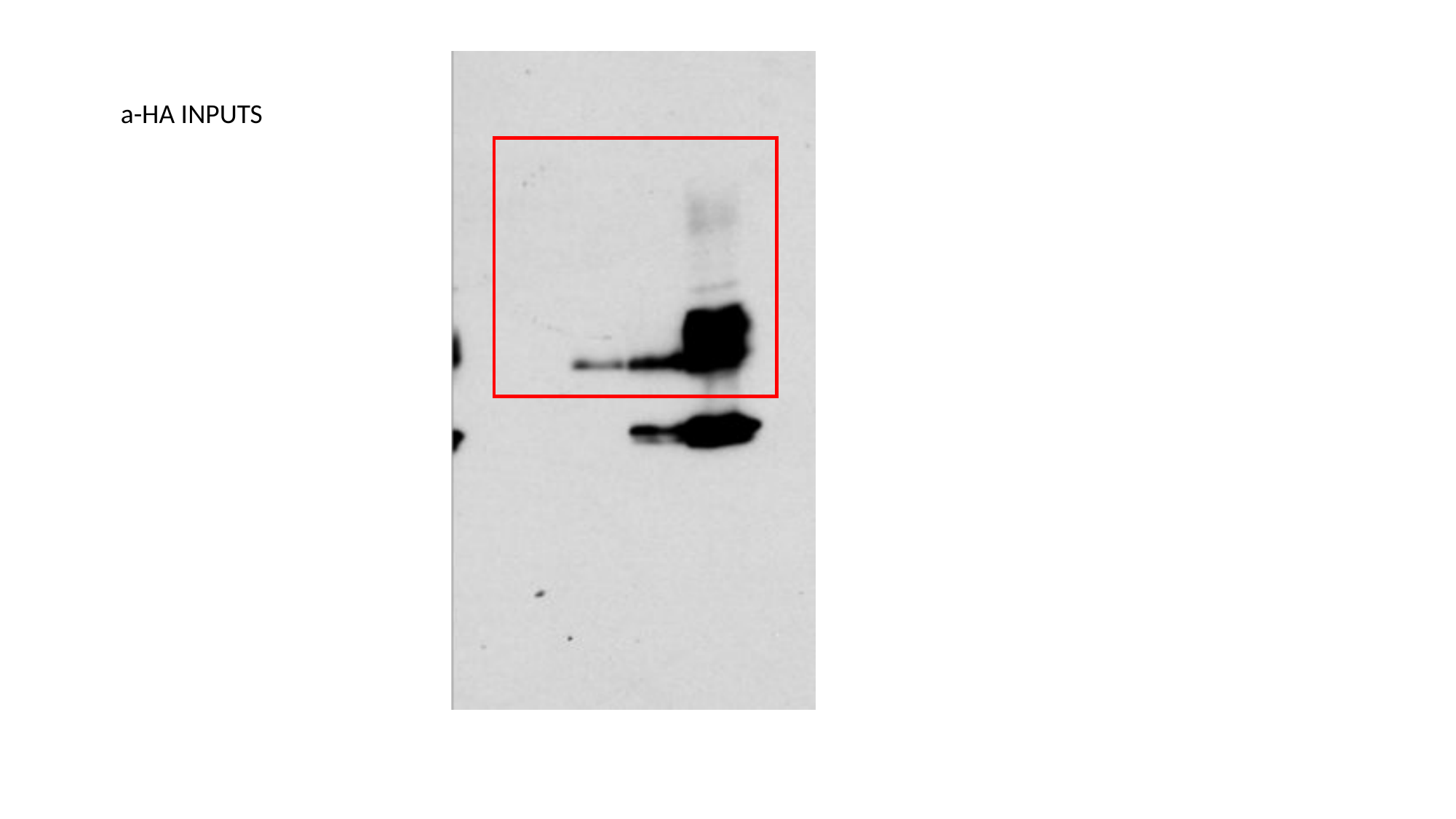

a-HA INPUTS

## Slide 2
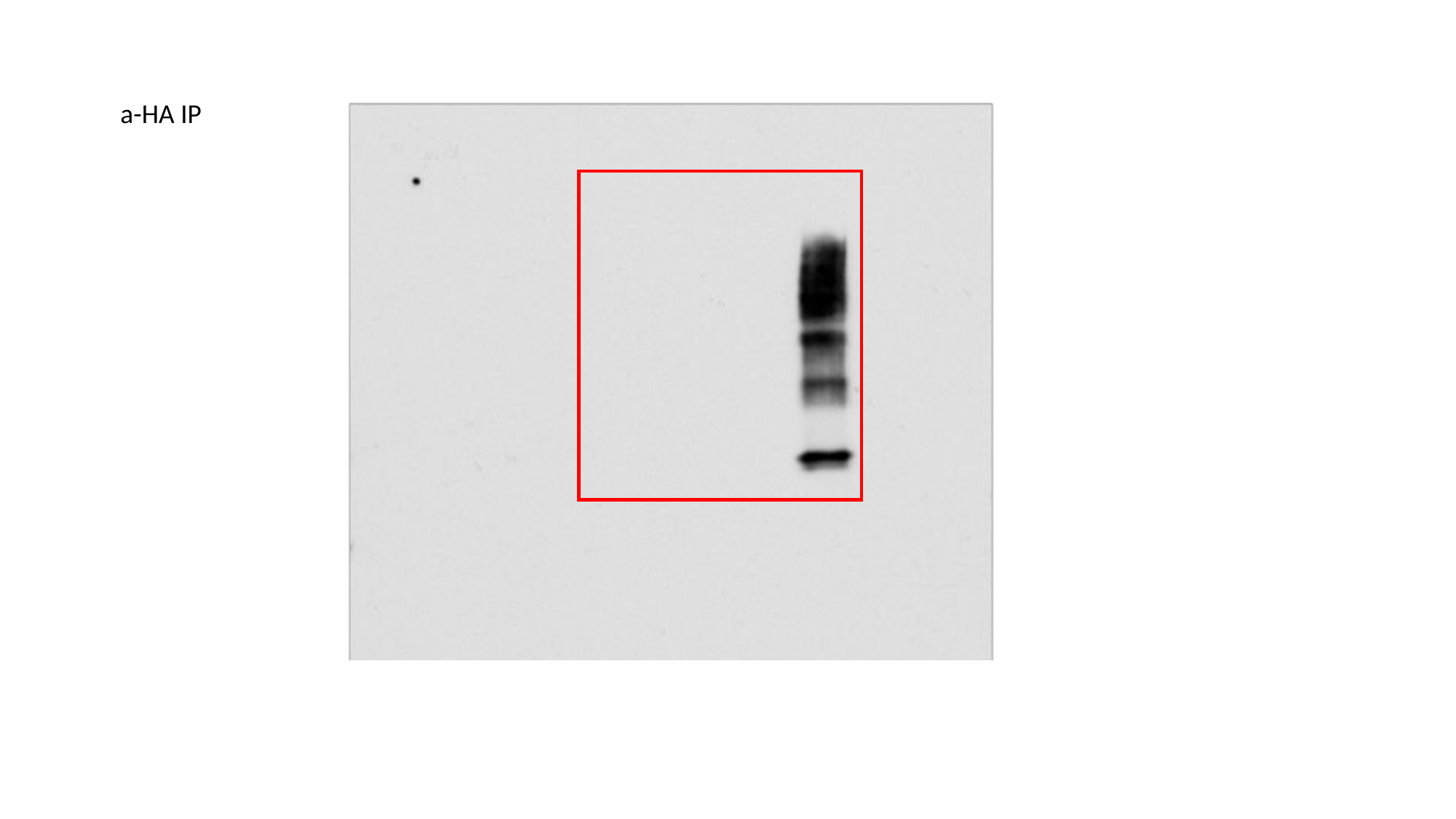

a-HA IP

Supplement: Supplementary file 10 — Source data Fig. 5 [file 44319_2025_566_MOESM10_ESM.zip › Fig 5/5D/5D WB CROPPING.pptx]

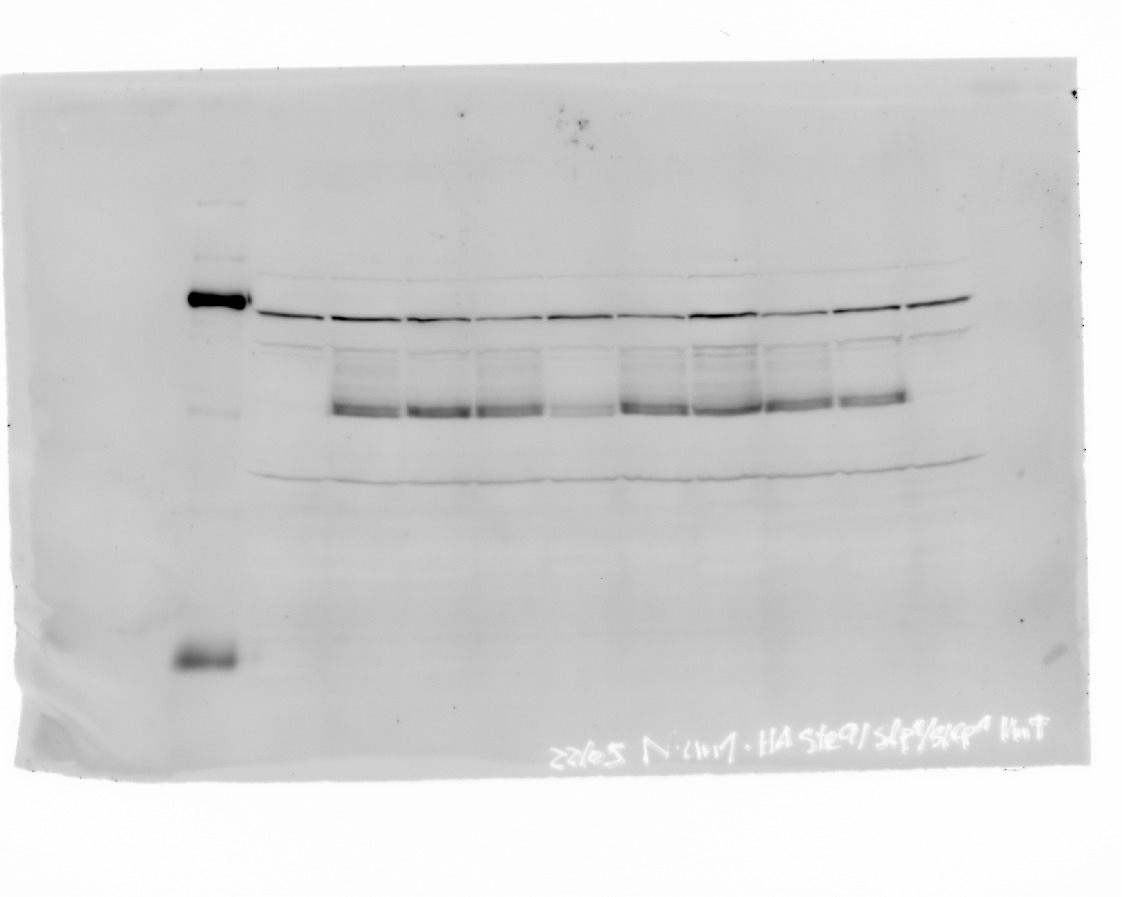

Supplement: Supplementary file 10 — Source data Fig. 5 [file 44319_2025_566_MOESM10_ESM.zip › Fig 5/5H/5H a-HA.tif]

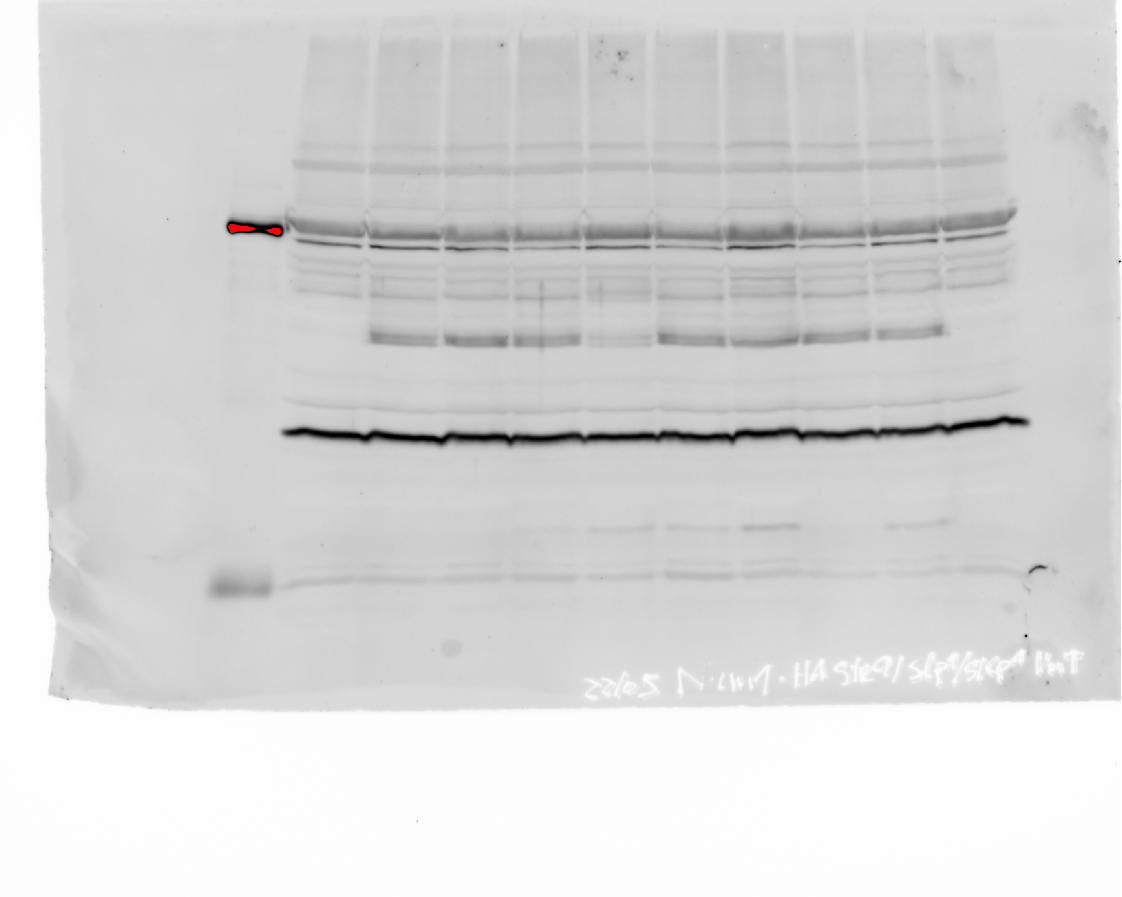

Supplement: Supplementary file 10 — Source data Fig. 5 [file 44319_2025_566_MOESM10_ESM.zip › Fig 5/5H/5H a-Sty1.tif]

## Slide 1
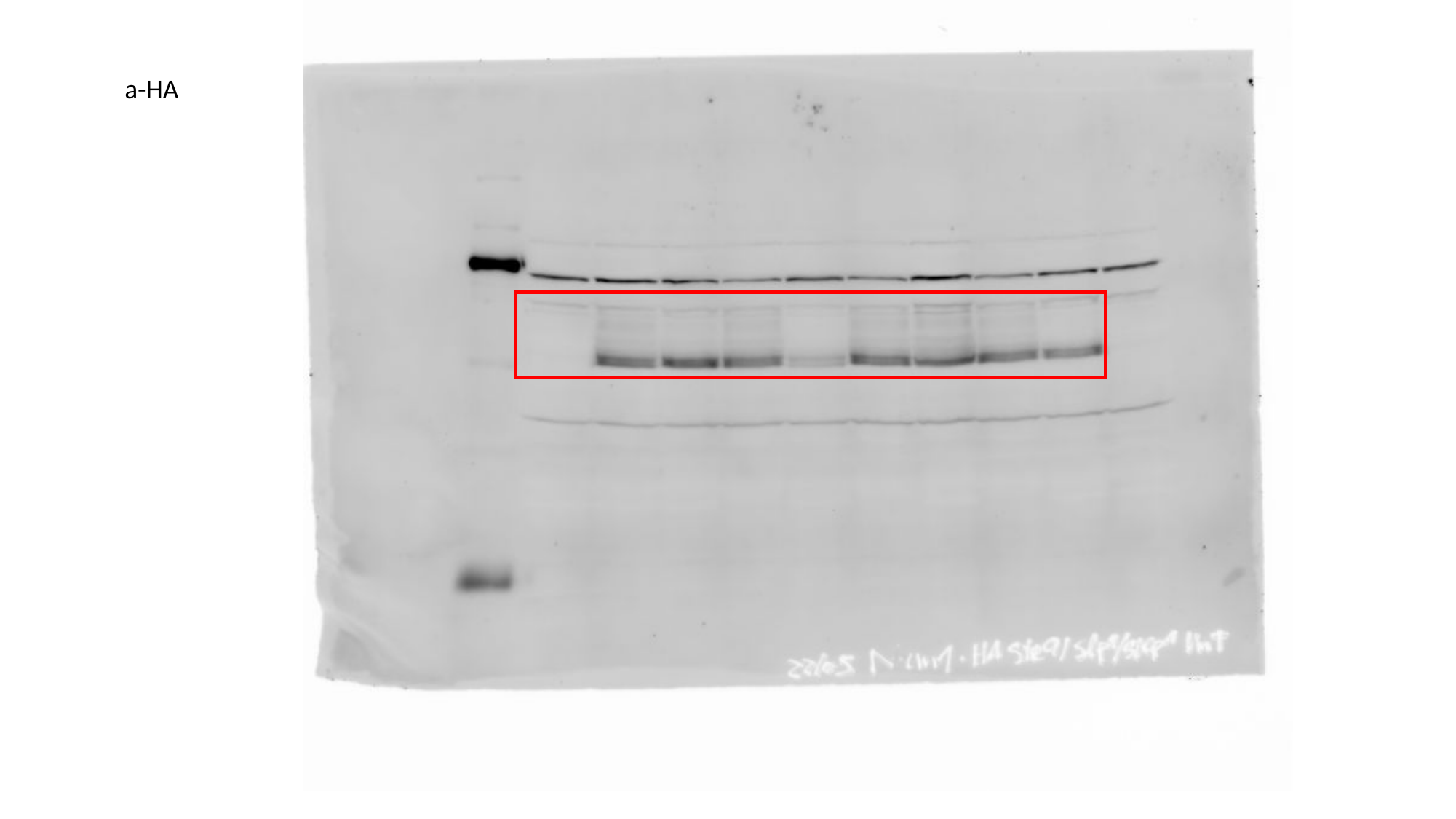

a-HA

## Slide 2
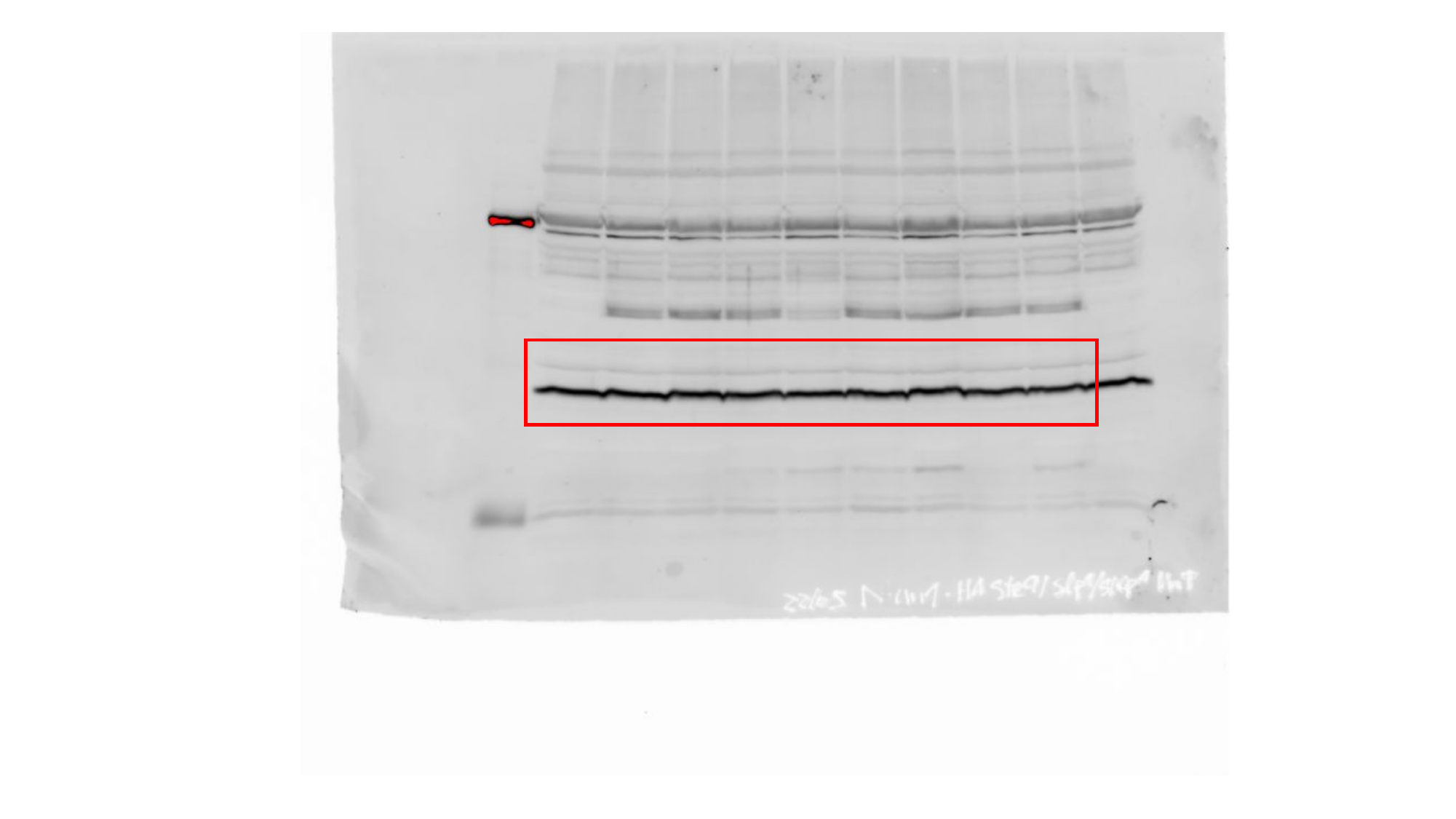

Supplement: Supplementary file 10 — Source data Fig. 5 [file 44319_2025_566_MOESM10_ESM.zip › Fig 5/5H/5H WB CROPPINGS.pptx]
