# Supplementary material for: Nrm1 is a bistable switch connecting cell cycle progression to transcriptional control
Source: EMBO Rep. 2025 Aug 29;26(20):5048–69. doi: 10.1038/s44319-025-00566-7 (PMC12550009; doi:10.1038/s44319-025-00566-7)
Supplement: Supplementary file 9 — Source data Fig. 4 [file 44319_2025_566_MOESM9_ESM.zip › Fig 4/4A/4A WB CROPPINGS.pptx]

## Slide 1
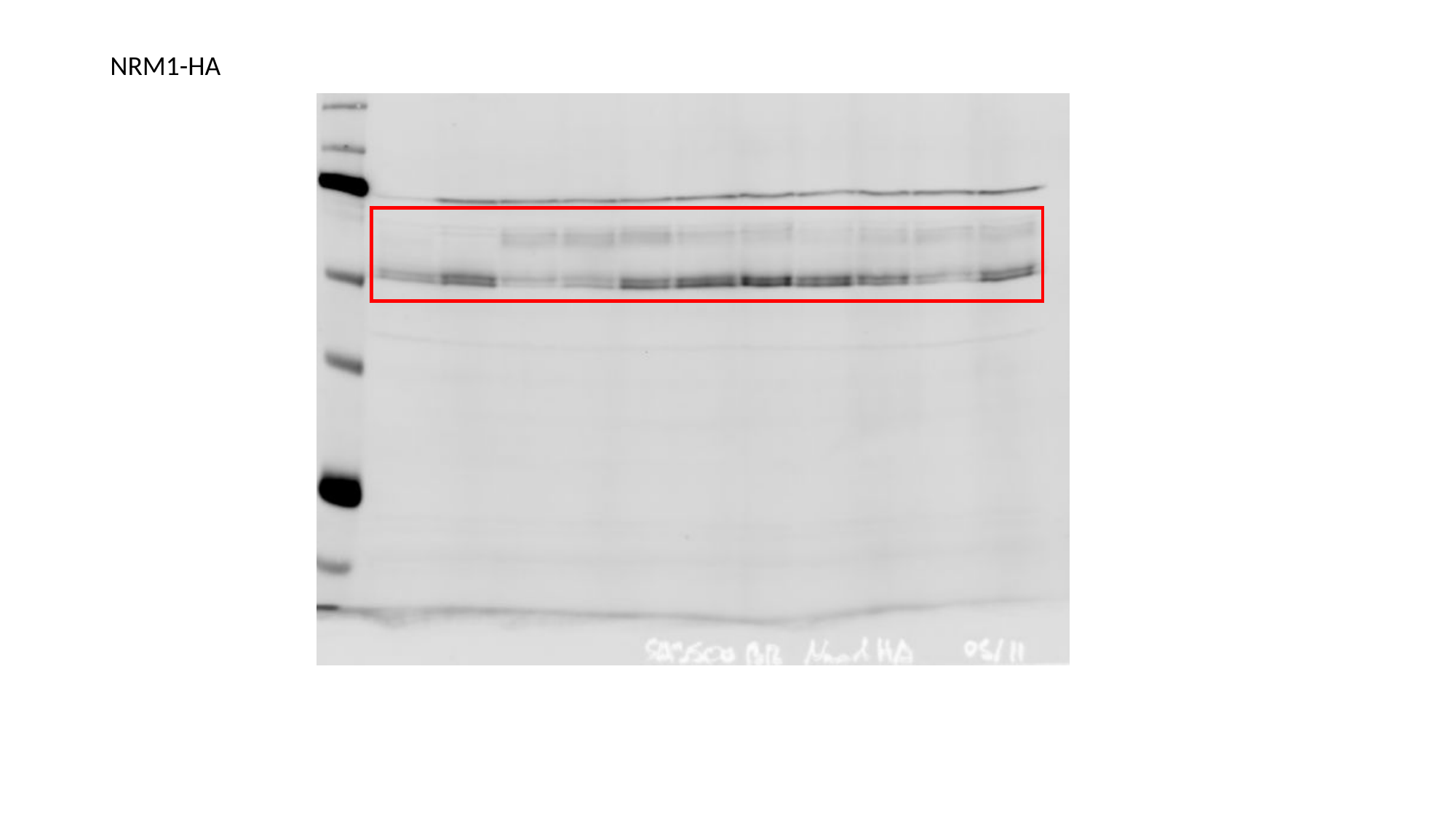

NRM1-HA

## Slide 2
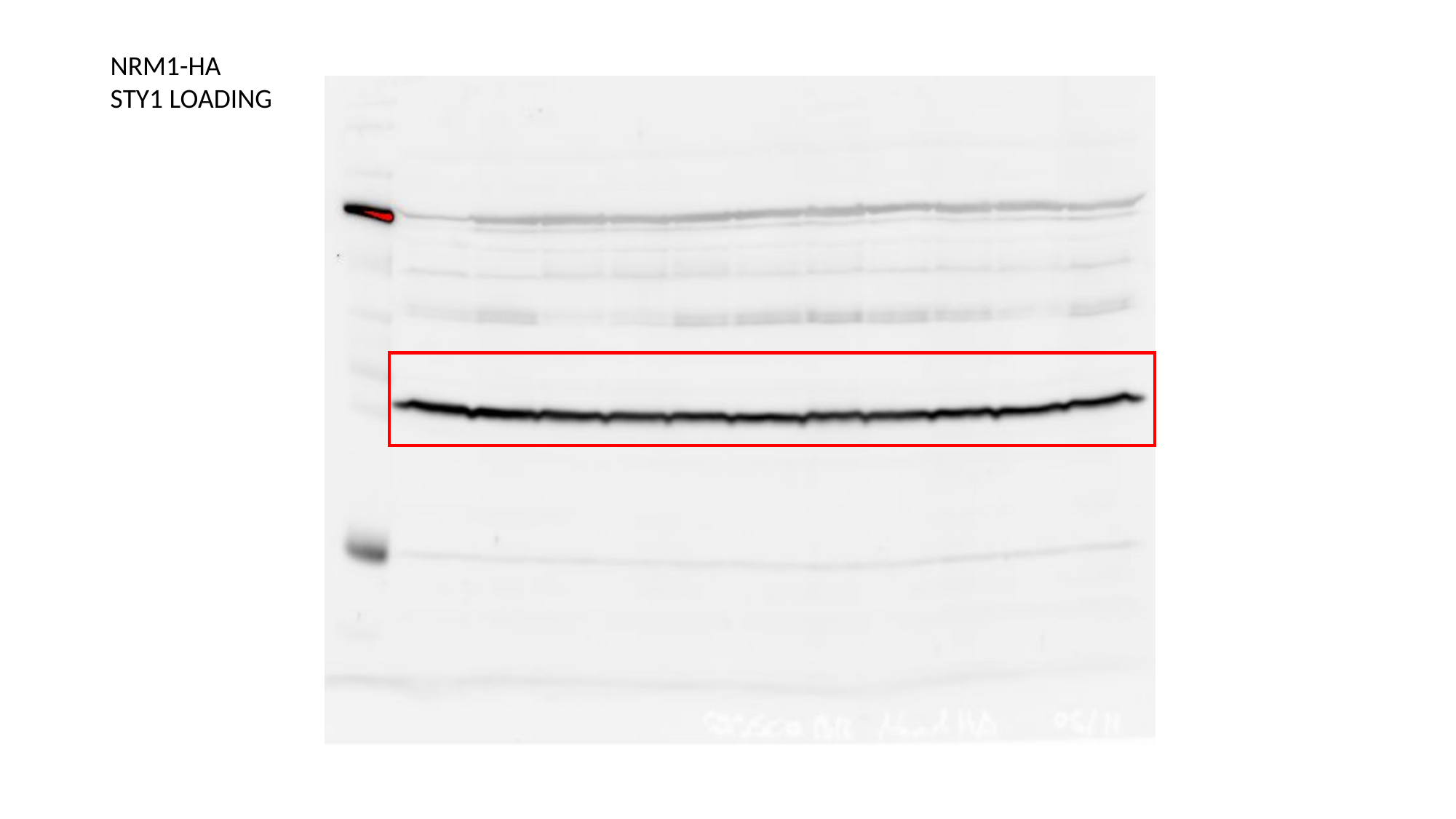

NRM1-HA
STY1 LOADING

## Slide 3
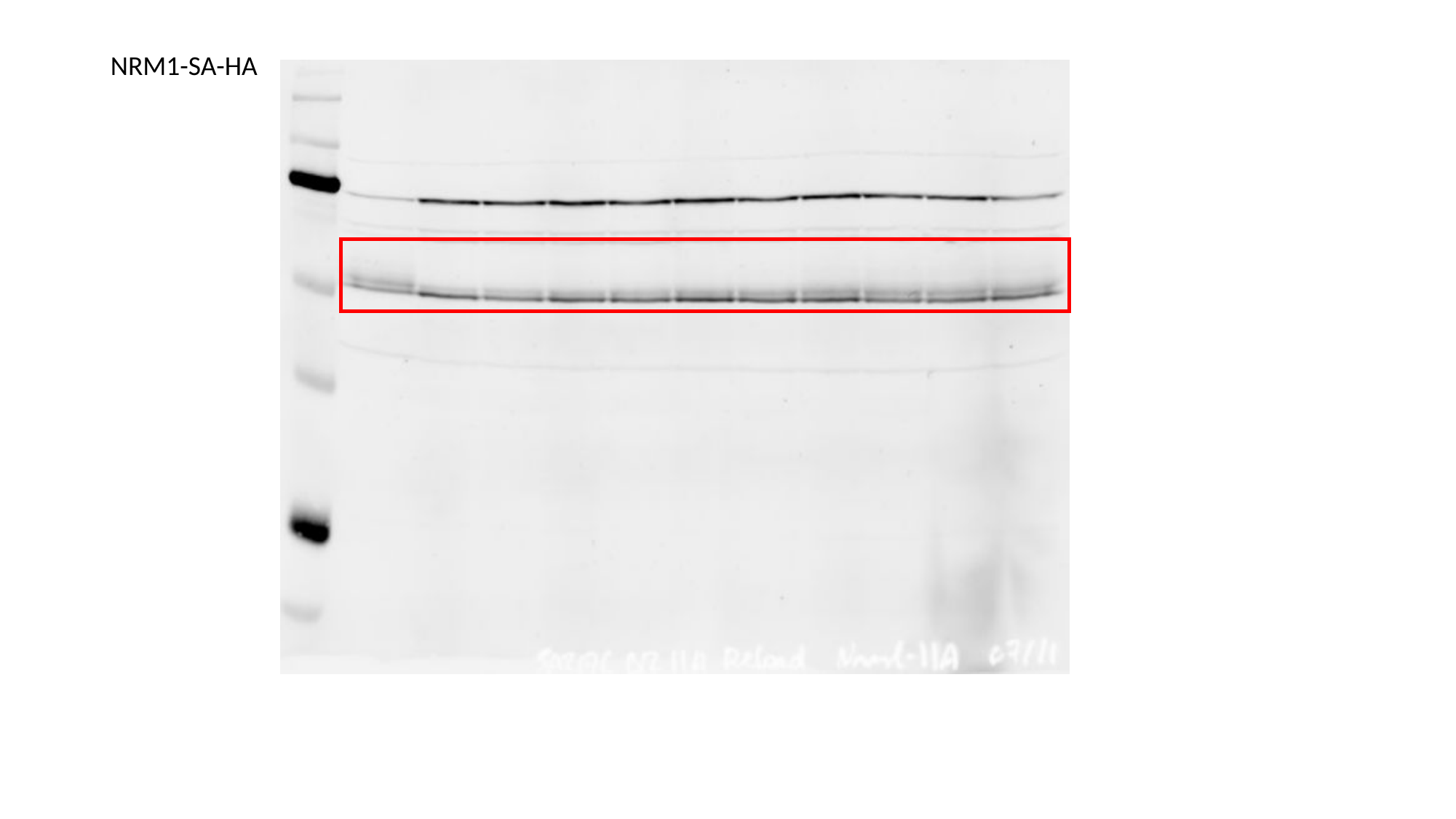

NRM1-SA-HA

## Slide 4
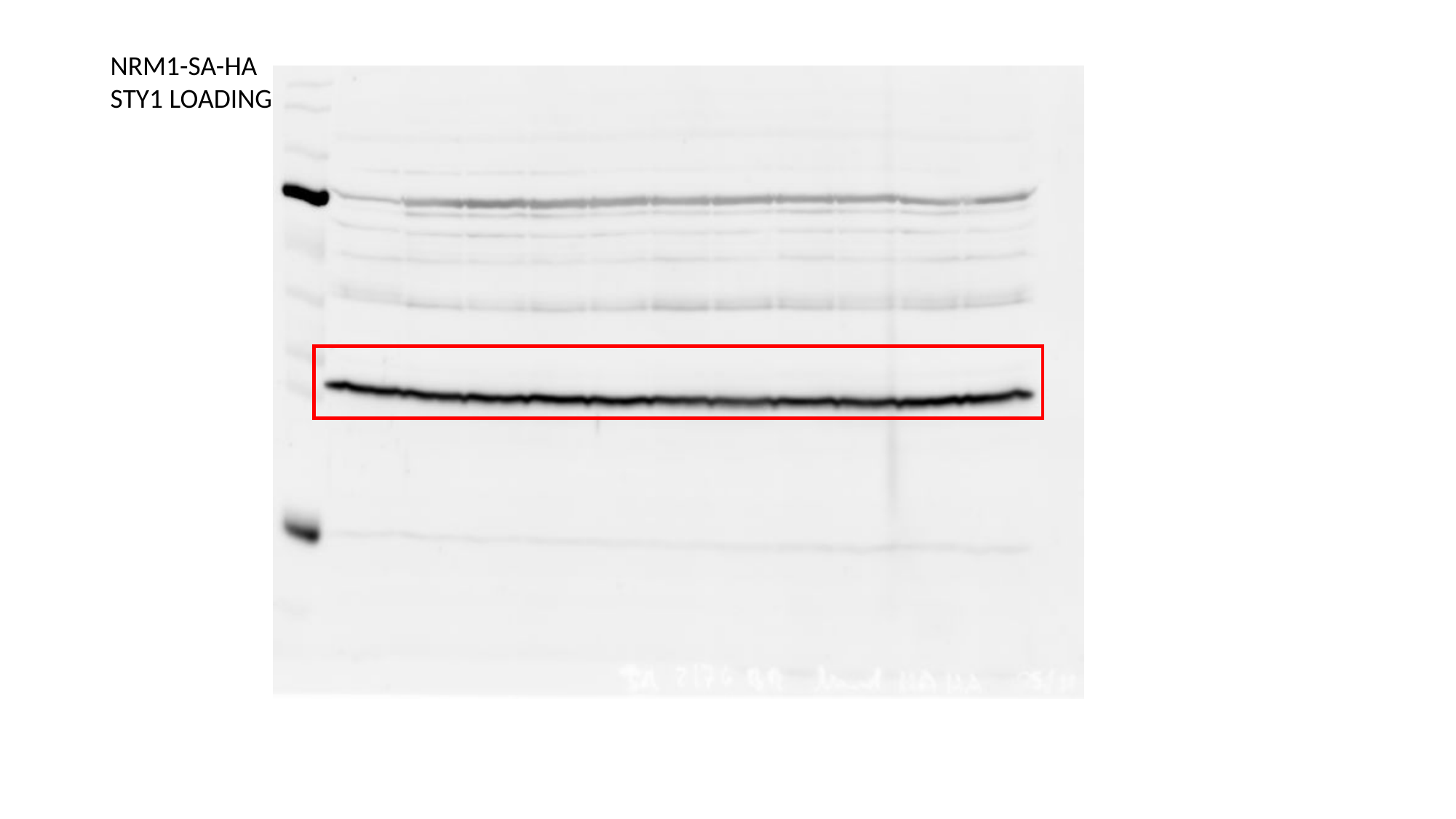

NRM1-SA-HA
STY1 LOADING

## Slide 5
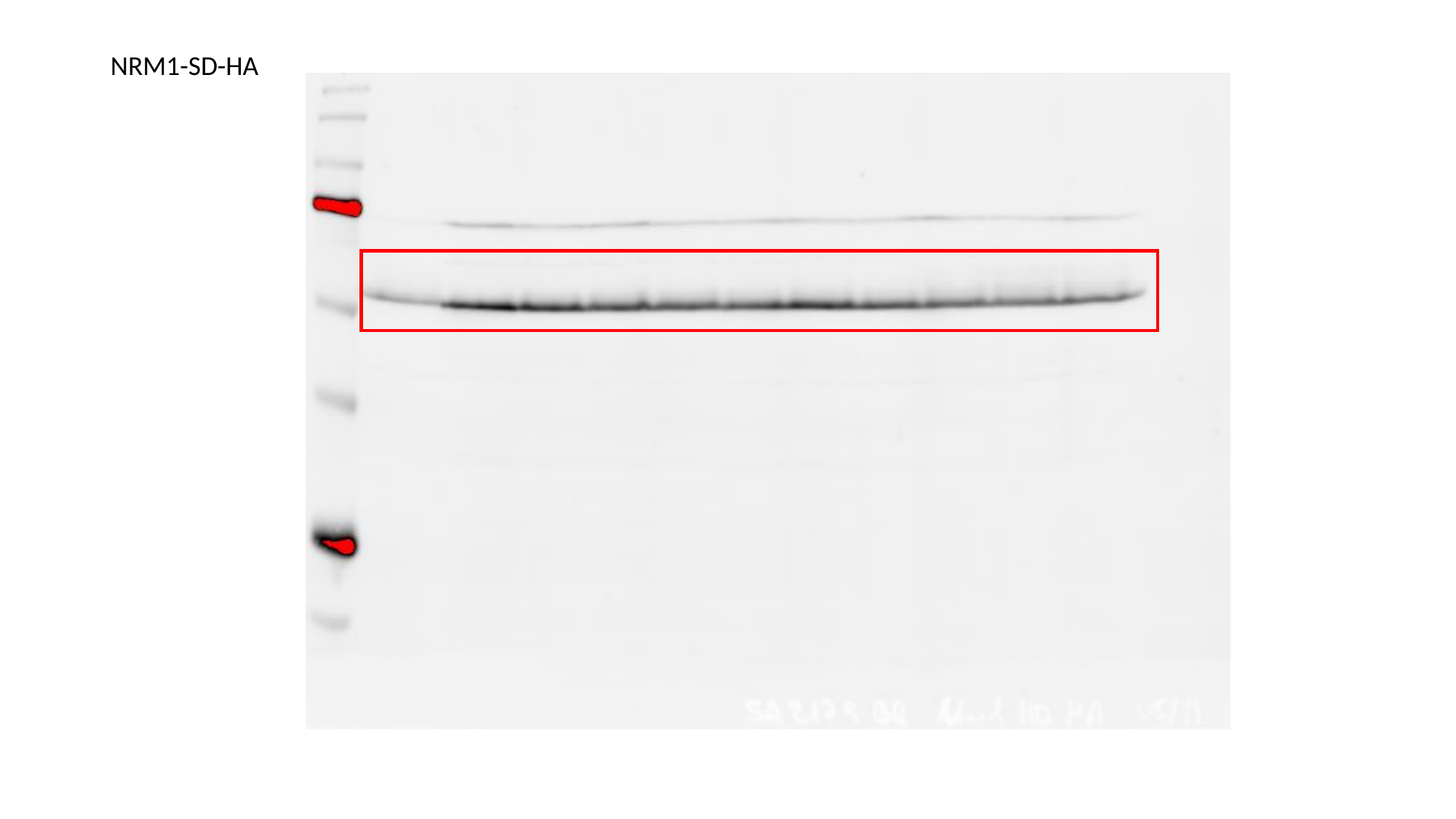

NRM1-SD-HA

## Slide 6
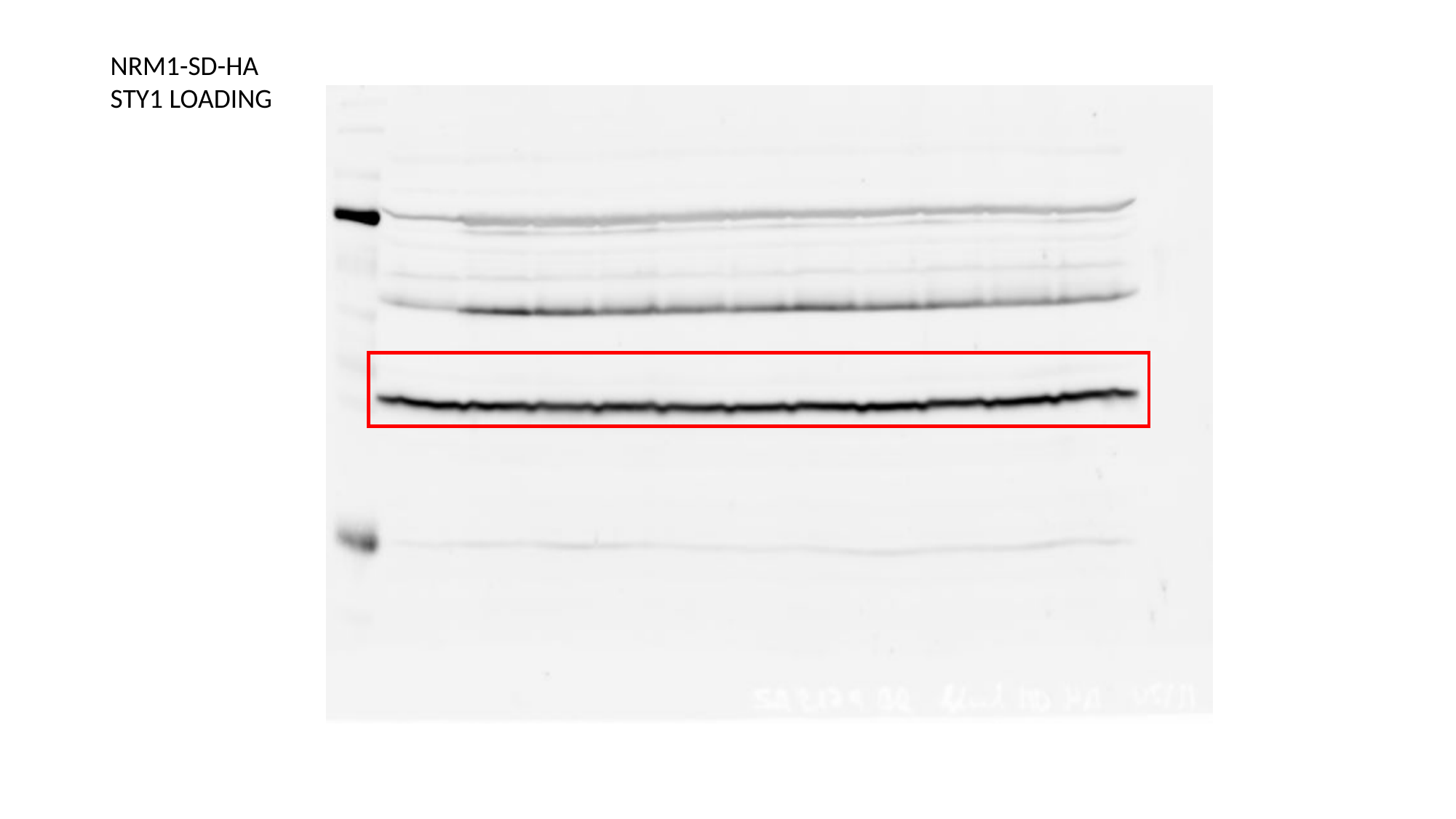

NRM1-SD-HA
STY1 LOADING
