# Supplementary material for: Nrm1 is a bistable switch connecting cell cycle progression to transcriptional control
Source: EMBO Rep. 2025 Aug 29;26(20):5048–69. doi: 10.1038/s44319-025-00566-7 (PMC12550009; doi:10.1038/s44319-025-00566-7)
Supplement: Supplementary file 12 — Expanded View Figures [file 44319_2025_566_MOESM12_ESM.pdf]

Expanded View Figures

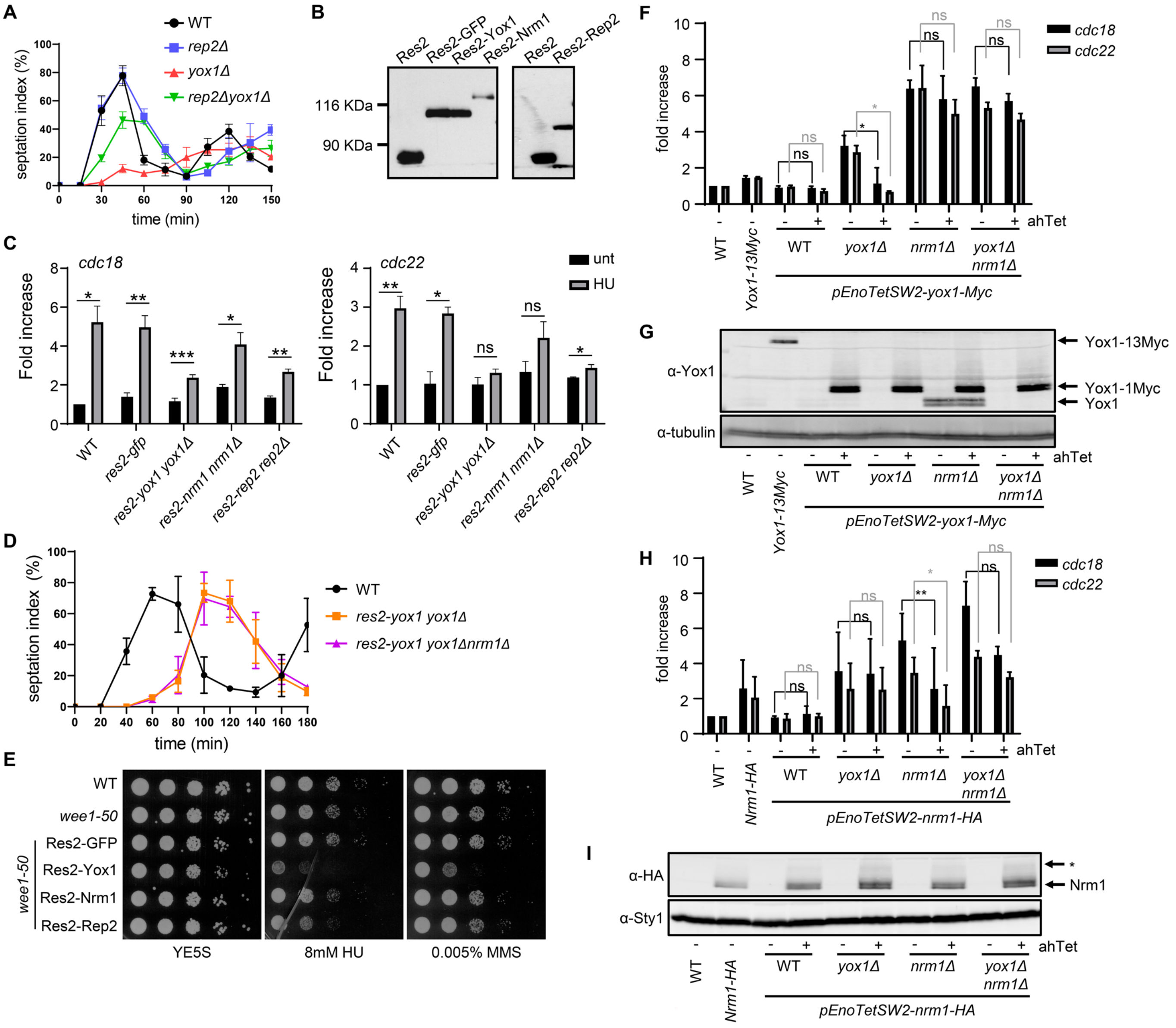

Figure EV1. Related to Fig. 1.

(**A**) Septation index of a wild type (WT, black), *rep2Δ* (blue), *yox1Δmik1Δ* (red) and *rep2Δyox1Δ* (green) in a *cdc2-asM17* block and release shown in Fig. 1B. Graphic represents mean ± SD of $n = 3$ experiments. (**B**) Western blot analysis of Res2 and Res2 chimeras. TCAs extracts were prepared from mid log cultures and separated in a 6% polyacrylamide gel. Western blot detection was performed with anti-HA antibodies. (**C**) qPCR of *cdc18* and *cdc22* expression in cultures grown in YE5S untreated (unt) or treated 3 h with 10 mM hydroxyurea (HU) of WT, *res2-gfp*, *res2-yox1 yox1Δ*, *res2-nrm1 nrm1Δ* and *res2-rep2 rep2Δ* cultures. *tfb2* was used as control gene. Expression was relativized to WT untreated. Graphic represents mean ± SD of $n = 3$ experiments. Statistics show significance from a Student's *T* test. ns: $p > 0.05$; *$p < 0.05$; **$p < 0.01$; ***$p < 0.001$. *p*(*cdc18* expression unt vs HU): 0.12325, 0.005623, 8.99E-06, 0.025819, 0.001346, respectively. *p*(*cdc22* expression unt vs HU): 0.008012, 0.020167, 0.179964, 0.142022, 0.026791. (**D**) Septation index of *cdc25-22* block and release experiment shown in Fig. 1D of WT (black), *res2-yox1 yox1Δ* (orange) *and res2-yox1 yox1Δnrm1Δ* (purple). Graphic represents mean ± SD of $n = 3$ experiments. (**E**) Survival was performed by spotting 10–105 cells of the indicated strains onto YE5S plates in the absence or presence of MMS or HU. Plates were incubated at 30 °C for 3–4 days. (**F, G**) qPCR of *cdc18* and *cdc22* (**F**) and α-Yox1 western blot (**G**) of WT, *yox1-13Myc* and WT, *yox1Δ*, *nrm1Δ and yox1Δnrm1Δ* strains expressing Yox1-1Myc under the control of a tetracycline (ahTet) inducible promoter *pEnoTetSW2*. *tfb2* was used as control gene in qPCRs and expression was then relativized to WT non-induced sample. Graphic represents mean ± SD of $n = 3$ experiments. Statistics show significance from a Student's *T* test. ns: $p > 0.05$; *$p < 0.05$; **$p < 0.01$; ***$p < 0.001$. *p*(*cdc18* expression unt vs ahTet): 0.9174, 0.0139, 0.5449 and 0.1358, respectively. *p*(*cdc22* expression unt vs ahTet): 0.0932, 0.0093, 0.03241 and 0.0929, respectively. In (**G**), it is shown one representative experiment out of three different experiments. α-Yox1 was used to detect endogenous and overexpressed Yox1. Tubulin is shown as a loading control. (**H, I**) Same experiment as in (**F, G**), but cells were expressing Nrm1-3HA under the control of a tetracycline (ahTet) inducible promoter *pEnoTetSW2*. In (**H**), *tfb2* was used as control gene in qPCRs and expression was then relativized to WT non-induced sample. Graphic represents mean ± SD of $n = 3$ experiments. Statistics show significance from a Student's *T* test. ns: $p > 0.05$; *$p < 0.05$; **$p < 0.01$; ***$p < 0.001$. *p*(*cdc18* expression unt vs ahTet): 0.5425, 0.9250, 0.0087 and 0.1104, respectively. *p*(*cdc22* expression unt vs ahTet): 0.6741, 0.9343, 0.0236 and 0.1170, respectively. In (**I**), one representative experiment is shown out of three different. α-HA was used to detect Nrm1-3HA expression. Sty1 is shown as a loading control.

© The Author(s)

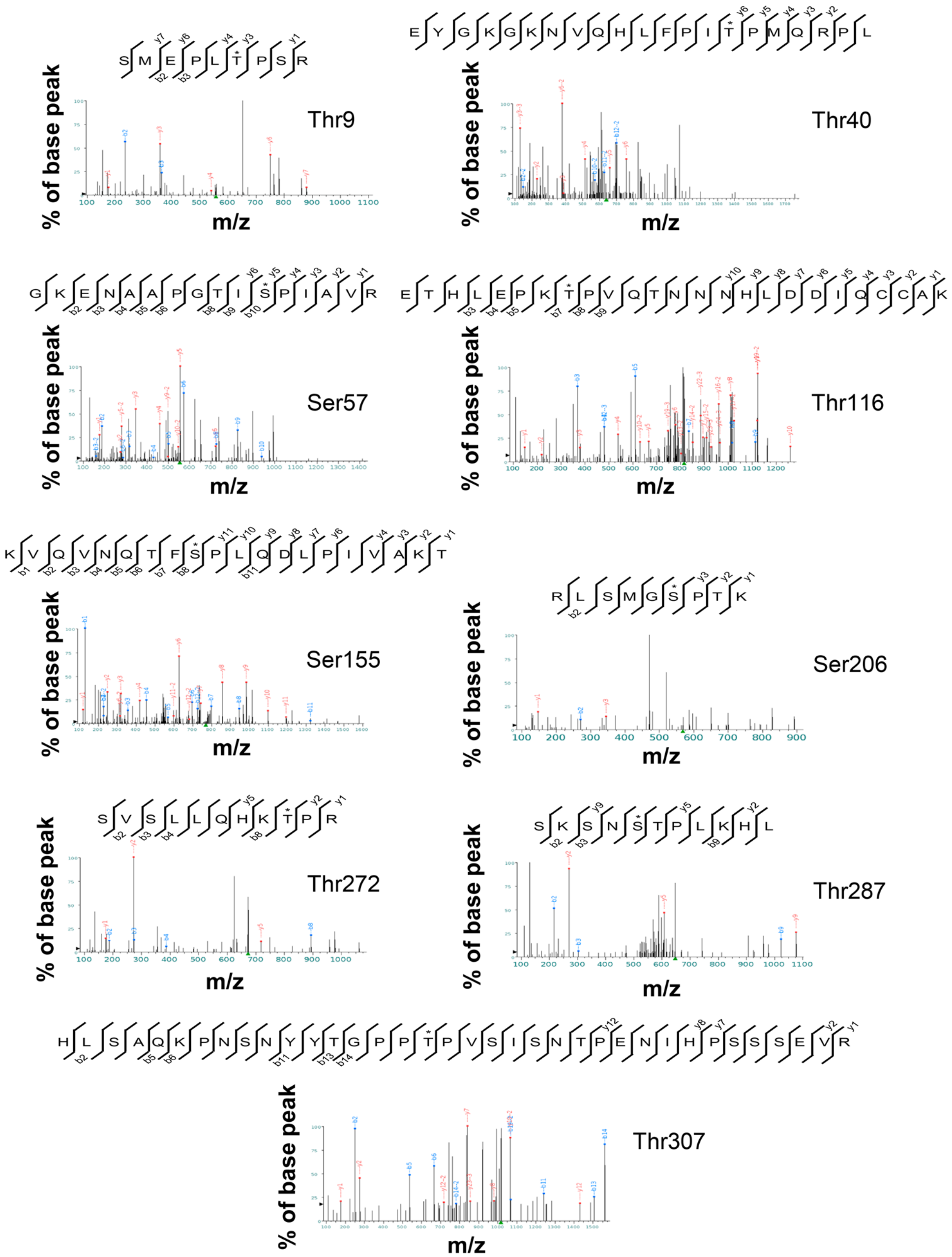

Figure EV2. Related to Fig. 3.

Original mass spectra map of putative phosphorylation sites in Nrm1. Purified Nrm1-HA from metaphase arrested cells was trypsin and/or chymotrypsin digested. The resulting peptides were analyzed by LC-MS/MS. The collision-induced dissociation MS2 and sequence of the phosphorylated peptides are shown.

© The Author(s)

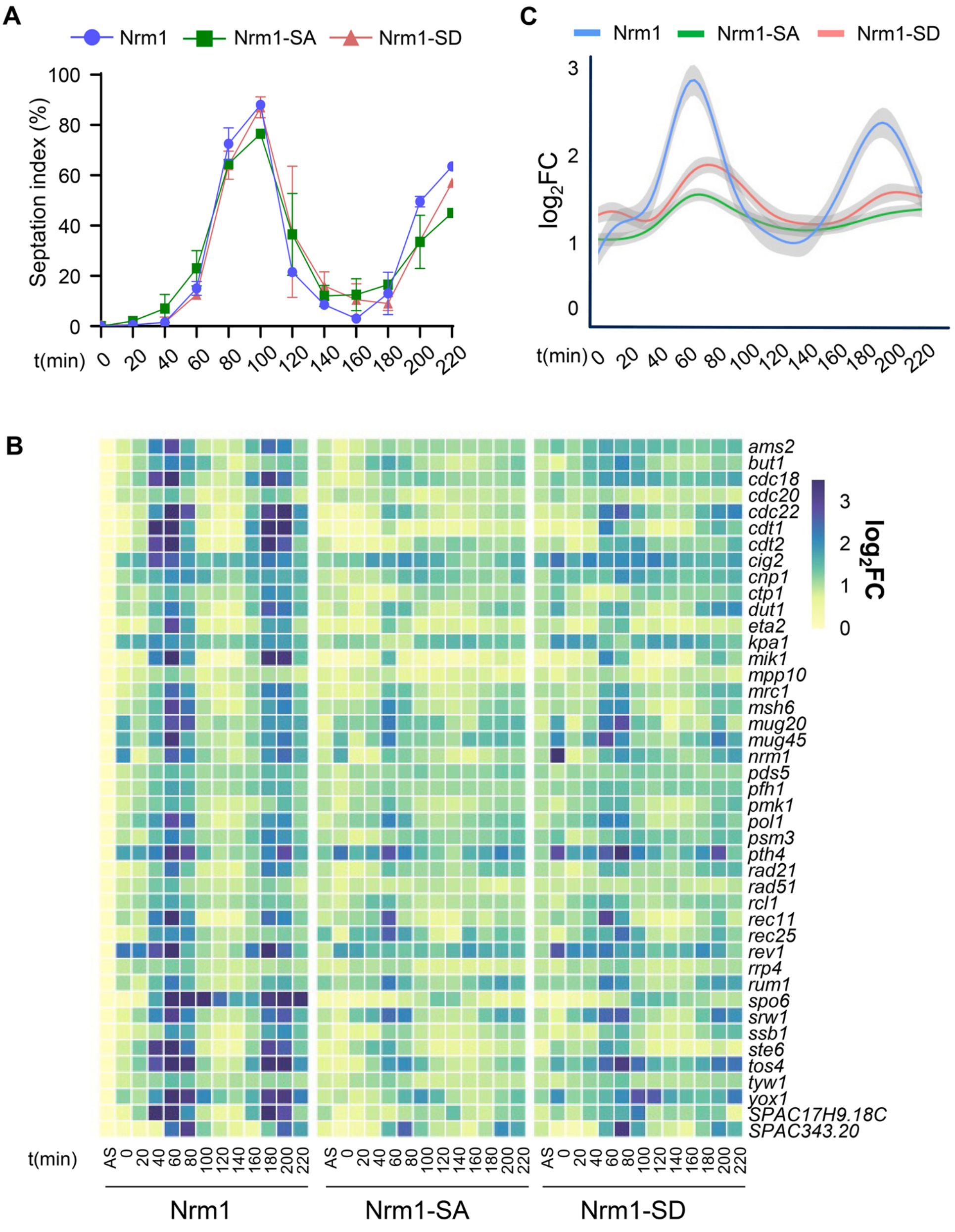

Figure EV3. Related to Fig. 4.

(**A**) Septation index of wild type (Nrm1, blue), Nrm1-SA (green) and Nrm1-SD (red) in a *cdc25-22* block and release shown in Fig. 4C,D. Graphic represents mean ± SD of $n = 3$ experiments. (**B**) Heatmap of the gene expression of 43 MBF-dependent genes. RNAseq of wild type (Nrm1), Nrm1-SA and Nrm1-SD in a *cdc25-22* block and release. Genes were selected as cycling genes with peaks of transcription at 60–80 and 180–200 min after release and having expression altered in Nrm1-SA and Nrm1 SD strains. Scale represents Log_2FC of normalized reads of each time-point relative to Nrm1 asynchronous (AS) values. (**C**) Expression of MBF-dependent genes from Fig. EV3B in a wild type (Nrm1, blue), Nrm1-SA (green) and Nrm1-SD (red). Line represents mean of values and grey shadow represents SD.

© The Author(s)

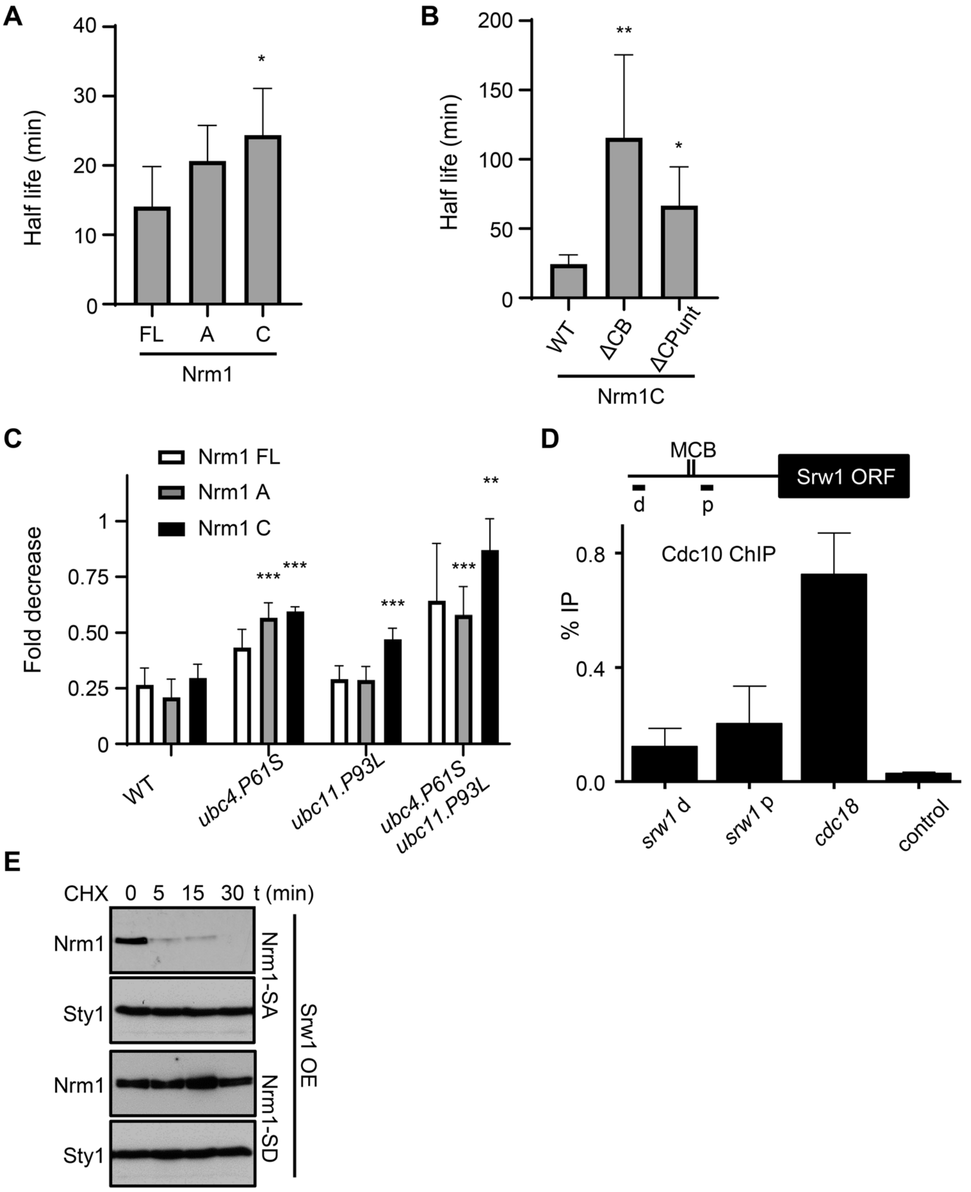

Figure EV4. Related to Fig. 5.

(**A**) Half-life in minutes of wild type full length Nrm1 (FL), the first half of Nrm1 containing the first 171 residues (**A**) and the carboxi-domain of Nrm1 containing the last 171 residues (**C**). TCA samples were collected at 0, 10, 30 and 60 min after cycloheximide addition and analyzed in western blot. Sample loading was normalized with Sty1. Half-life was calculated using GraphPad Prism. Plot represents mean ± SD of at least $n = 3$ experiments. Statistics show significance from a Student's *T* test. $^*p < 0.05$; $^{**}p < 0.01$; $^{***}p < 0.001$. *p*(Nrm1 FL vs Nrm1 A): 0.0641. p(Nrm1 FL vs Nrm1 C): 0.0232. (**B**) Half-life in minutes of the carboxi-domain of Nrm1 containing the last 171 residues (Nrm1 C) native sequence (WT), or with a deletion of the CBox (ΔCB) or with 5 amino acid substitutions (H252A W255A, R263A, V265A, L267A) (ΔCPunt). TCA samples were collected at 0, 10, 30 and 60 min after cycloheximide addition and analyzed in western blot. Sample loading was normalized with Sty1. Half-life was calculated using GraphPad Prism. Plot represents mean ± SD of at least $n = 3$ experiments. Statistics show significance from a Student's *T* test. $^*p < 0.05$; $^{**}p < 0.01$; $^{***}p < 0.001$. *p*(Nrm1 C WT vs ΔCB): 0.0096. p(Nrm1 C WT vs ΔCB): 0.0149. (**C**) Protein fold change (FC) of Nrm1 full length (Nrm1-FL), Nrm1-A and Nrm1-C in wild type (WT), *ubc4.P61S*, *ubc11.P93L*, and *ubc4.P61S ubc11.P93L* strains. Cells were grown at 25 °C and shifted at 37 °C for 1 h before adding cycloheximide. Samples were collected before and 1 h after the treatment. TCA extracts were performed and assayed by western blot. Loading was normalized with Sty1. Plot represents mean ± SD of at least $n = 3$ experiments. Statistics show significance from a Student's *T* test. $^*p < 0.05$; $^{**}p < 0.01$; $^{***}p < 0.001$. *p*(Nrm1 FL vs WT): 0.06, 0.666 and 0.0717, respectively. *p*(Nrm1 A vs WT): 2.44E-05, 0.1107 and 0.0004, respectively. *p*(Nrm1 C vs WT): 3.9E-05, 0.0064 and 0.0001, respectively. (**D**) Cdc10 Chip from asynchronous cultures; *srw1* d, distal region of *srw1* promoter; *srw1* p, proximal region of *srw1* promoter. On top, a scheme of *srw1*, indicating the 2 MCB sites in the promoter; d, distal amplicon; p, proximal amplicon. Plot represents mean ± SD of at least $n = 3$ experiments. Statistics show significance from a Student's *T* test. $^*p < 0.05$; $^{**}p < 0.01$; $^{***}p < 0.001$. (**E**) Western blot of extracts prepared from wild type cells overexpressing Srw1 (Srw1 OE). Cells were expressing endogenous Nrm1-SA or Nrm1-SD. On top, time after the addition of cycloheximide (CHX). Sty1 is shown as loading control. One representative experiment is shown out of three different experiments.

 © The Author(s)

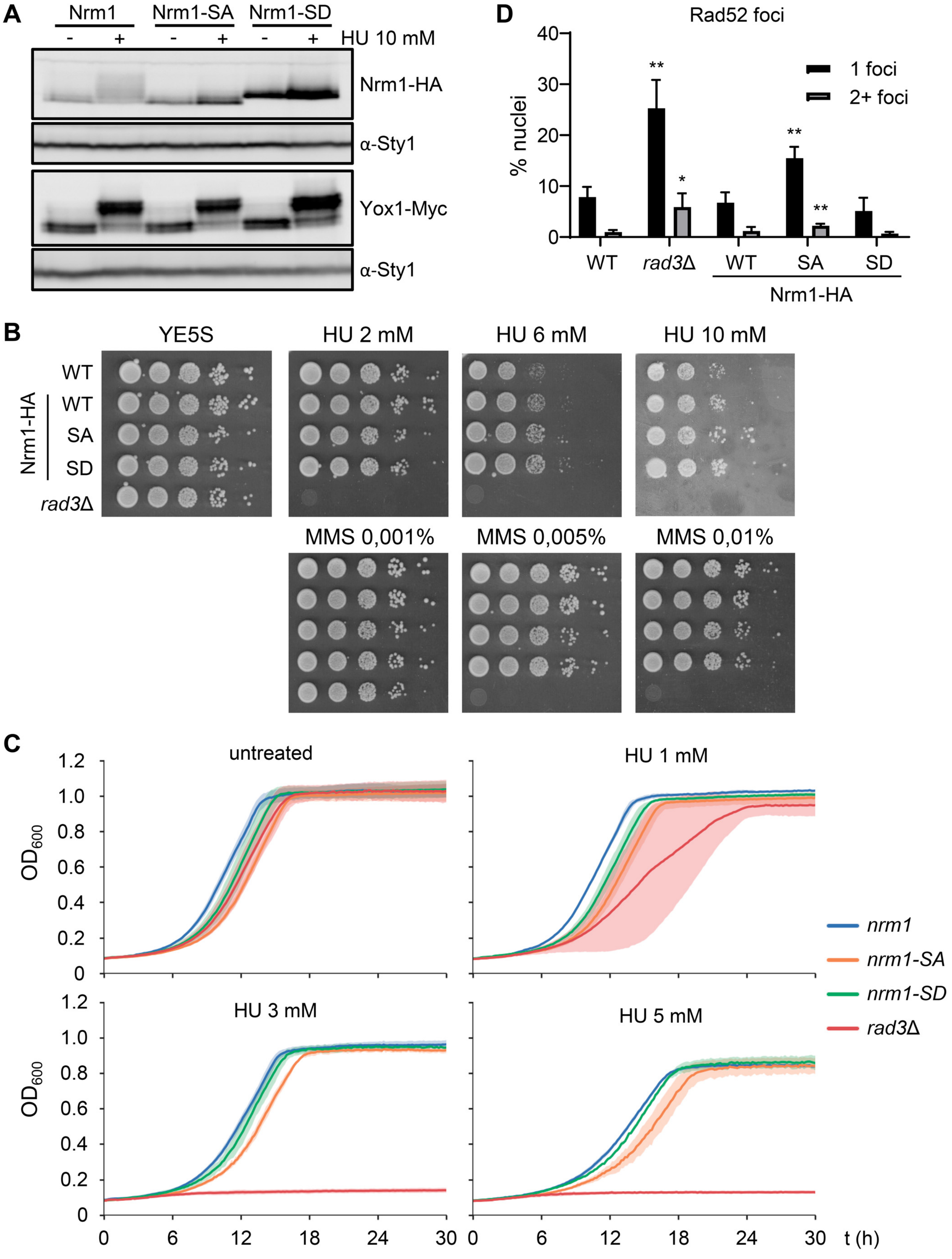
A
Nrm1
Nrm1-SA
Nrm1-SD
- + - + - + HU 10 mM
Nrm1-HA
α-Sty1
Yox1-Myc
α-Sty1
D
Rad52 foci
% nuclei
1 foci
2+ foci
WT rad3Δ WT SA SD
Nrm1-HA
B
YE5S
HU 2 mM
HU 6 mM
HU 10 mM
WT
Nrm1-HA
WT
SA
SD
rad3Δ
MMS 0,001%
MMS 0,005%
MMS 0,01%
C
untreated
HU 1 mM
HU 3 mM
HU 5 mM
OD600
t (h)
nrm1
nrm1-SA
nrm1-SD
rad3Δ

Figure EV5. Related to Fig. 6.

(**A**) Western blot analysis of Nrm1 and Yox1 in strains with wild type Nrm1, Nrm1-SA or Nrm1-SD. Cells were collected before and after treatment with hydroxyurea (HU) 10 mM for 3 h. Sty1 is shown as loading control. One representative experiment is shown out of three different replicas. (**B**) Survival was performed by spotting 10–10^5 cells of the indicated strains onto YE5S plates in the absence or presence of the indicated concentrations of MMS or HU. Plates were incubated at 30 °C for 3–4 days. (**C**) Growth curves comparing growth at 30 °C of the indicated strains in YE5S in the absence or presence of different concentrations of hydroxyurea (HU). Plot represents mean ± SD of at least $n = 3$ experiments. (**D**) Percentage of nuclei containing Rad52-mNeonGreen foci in WT, *rad3Δ, nrm1-HA, nrm1-SA-HA* and *nrm1-SD-HA* strains grown in YE5S. At least 100 nuclei were counted per replica. Plot represents mean ± SD of at least $n = 3$ experiments. Statistics show significance from a Student's *T* test. $*p < 0.05$; $**p < 0.01$. p(1 foci vs WT): 0.0010, 0.4701, 0.0051 and 0.1745, respectively. p(2+ foci vs WT): 0.0121, 0.6422, 0.0099 and 0.3624, respectively.

© The Author(s)
